# Ultrasound‐Activated Piezoelectric Neuroimmune Hydrogel Orchestrates Neurogenesis‐Macrophage Crosstalk in Diabetic Wound Healing

**DOI:** 10.1002/advs.76721

**Published:** 2026-07-28

**Authors:** Kai Wang, Shaowen Zhuo, Binyu Song, Jinan Chen, Xin Zhao, Sijia Li, Shuang You, Dong Jiang, Yuedong Chen, Juanli Dang, Tong Wang, Baolin Guo, Zhou Yu, Baoqiang Song

**Affiliations:** ^1^ Department of Plastic Surgery Xijing Hospital The Fourth Military Medical University Xi'an China; ^2^ Diabetic Foot Center The Air Force Hospital of Eastern Theater of PLA Nanjing China; ^3^ State Key Laboratory For Mechanical Behavior of Materials, and Frontier Institute of Science and Technology Xi'an Jiaotong University Xi'an China; ^4^ State Key Laboratory of Oral & Maxillofacial Reconstruction and Regeneration The Fourth Military Medical University Xi'an China

**Keywords:** diabetic wound healing, macrophage reprogramming, mitochondrial dysfunction modulation, neuroimmune crosstalk, ultrasound‐activated piezoelectric hydrogel

## Abstract

Chronic diabetic wounds remain a major clinical challenge due to persistent inflammation, impaired neuro‐immune crosstalk, and dysregulated macrophage polarization. Here, we report an injectable, ultrasound (US)‐activated piezoelectric hydrogel that programmably couples neurogenesis and macrophage reprogramming to accelerate diabetic wound repair. The hydrogel is constructed from dynamic covalent networks among acyl hydrazide hyaluronic acid, benzene boronic acid modified oxidized sodium alginate, and epigallocatechin 3‐gallate, and integrates a neuroactive self‐assembling peptide and piezoelectric PLLA nanofibers. This design confers the hydrogel with injectability, self‐healing, tissue adhesion, and US‐triggered electrical signaling. In vitro, the hydrogel under US promotes M2 macrophage polarization and enhances axonal outgrowth of dorsal root ganglion neurons, establishing bidirectional neurogenesis‐macrophage crosstalk. In a type II diabetic mouse model, the hydrogel markedly accelerates wound closure, elevates the M2/M1 ratio, enhances reinnervation and angiogenesis, and improves collagen remodeling. Transcriptomic analysis of cells and wound tissues identifies coordinated regulation of mitochondrial function and oxidative phosphorylation as a central mechanism. In a diabetic rabbit ear model, the hydrogel with US further attenuates hypertrophic scar formation. This programmable piezoelectric hydrogel converts noninvasive US into spatially confined bioelectrical and biochemical cues, offering a promising strategy to overcome multifactorial barriers in diabetic wound healing.

## Graphical abstract

In this work, we design an ultrasound‐activated piezoelectric hydrogel platform (AOEF@AP5+US) that reprograms neurogenesis‐macrophage crosstalk as an integrated strategy to overcome the intertwined barriers of chronic inflammation, peripheral neuropathy, and impaired regeneration in diabetic wounds. This programmable piezoelectric hydrogel converts noninvasive US into spatially confined bioelectrical and biochemical cues, offering a promising strategy to overcome multifactorial barriers in diabetic wound healing.

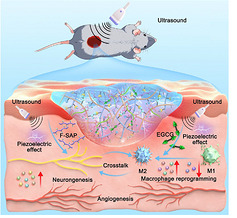

## 1 Introduction

Diabetic wounds constitute a rapidly escalating global health burden, arising from the combined effects of peripheral neuropathy, vascular insufficiency, and immune dysregulation [1, 2]. These factors synergistically lead to chronic, non‐healing ulcers that severely impair quality of life and dramatically increase the risk of infection and amputation [3]. Clinically used therapies largely target isolated aspects of pathology, while failing to address the multi‐layered neurovascular and immune defects that underpin impaired healing [4, 5]. Therefore, there is an urgent need for advanced therapeutic strategies that move beyond symptomatic management and actively reprogram the pathological microenvironment of diabetic wounds.

Peripheral neuropathy diminishes sensory innervation and neuropeptide release in diabetic wounds, while macrophage function is skewed toward a pro‐inflammatory M1 phenotype, together locking wounds in a state of chronic inflammation [6, 7, 8, 9, 10, 11, 12]. Reprogramming macrophages toward an anti‐inflammatory, pro‐regenerative M2 phenotype is thus a key objective in restoring a pro‐healing milieu [13, 14, 15]. Emerging evidence indicates sensory neurons, and their neuropeptides modulate macrophage recruitment, phenotype, and effector functions [16, 17, 18, 19]. For example, calcitonin gene‐related peptide (CGRP)–positive sensory fibers and sensory‐derived TAFA4 have been shown to orchestrate macrophage‐mediated resolution of inflammation and tissue regeneration [18, 19]. However, in diabetic wounds, hyperglycemia, neuropathy, and macrophage dysfunction reinforce one another, creating a vicious cycle that current therapies fail to break [11, 20, 21]. Therapeutic platforms that can simultaneously restore neurogenesis and recalibrate macrophage polarization, thereby rebuilding neuron–macrophage crosstalk, remain largely unexplored [22, 23, 24, 25, 26].

Exogenous electrical stimulation (ES) has emerged as a powerful upstream regulator of wound repair, exerting coordinated effects on macrophages, peripheral nerves, endothelial cells, and fibroblasts [27, 28, 29, 30, 31, 32, 33]. ES can support neuronal survival and axonal outgrowth [34], bias macrophages toward an M2‐like phenotype [35, 36, 37], and promote angiogenesis and matrix remodeling. These observations suggest that spatiotemporally controlled bioelectric cues could act as an initiating signal to synchronize neurogenesis and macrophage reprogramming, establishing a positive feedback loop between nerve regeneration and immune modulation that accelerates skin tissue repair. However, conventional ES typically requires wired electrodes or implanted devices, which limit its precision, spatial adaptability, and clinical practicality in chronic wounds.

Piezoelectric biomaterials offer a minimally invasive route to generate ES in situ by converting mechanical energy, such as ultrasound (US)–induced acoustic pressure, into local electrical signals [38]. Their regenerative potential has been investigated in bone, cartilage, skeletal muscle, skin, and neural tissues [32, 39]. Classical piezoelectric polymers such as poly(vinylidene fluoride) (PVDF) can promote myogenic behavior through surface charge–mediated interactions [40], and BaTiO_3_‐based piezoelectric hydrogels have shown promise in skin repair [41]. Nonetheless, many inorganic or fluorinated piezoelectric face translational hurdles related to non‐degradability, limited biosafety, or complex processing [42, 43]. By contrast, poly (L‐lactic acid) (PLLA) is an FDA‐approved, biodegradable piezoelectric polymer widely utilized in tissue scaffolds, making it an attractive candidate for clinically oriented bioelectric interfaces [44]. Solid‐state PLLA nanofiber scaffolds have been reported to generate electric charges under US stimulation and promote bone and cartilage regeneration [45], yet their stable dispersion in soft hydrogels and systematic application in skin and neuroimmune regulation are far from fully established.

In addition, complementary biochemical cues can be harnessed to simultaneously target neurogenic and immunologic defects. Functional self‐assembling peptides (F‐SAP) have demonstrated potent effects in promoting spinal cord and peripheral nerve repair by providing bioactive matrices that guide axonal extension and neural network reconstruction [46]. In parallel, the natural polyphenol epigallocatechin‐3‐gallate (EGCG) has been shown to bias macrophages toward an anti‐inflammatory M2 phenotype and exhibit robust antioxidant and anti‐inflammatory activities [47]. We therefore hypothesized that integrating US‐activated PLLA‐based piezoelectric nanofibers with F‐SAP–mediated neuroregeneration and EGCG‐driven immunomodulation within a single hydrogel platform could re‐establish neuron–macrophage crosstalk and disrupt the chronic inflammatory‐denervation loop of diabetic wounds, an approach that, to our knowledge, has not yet been reported.

**SCHEME 1:**
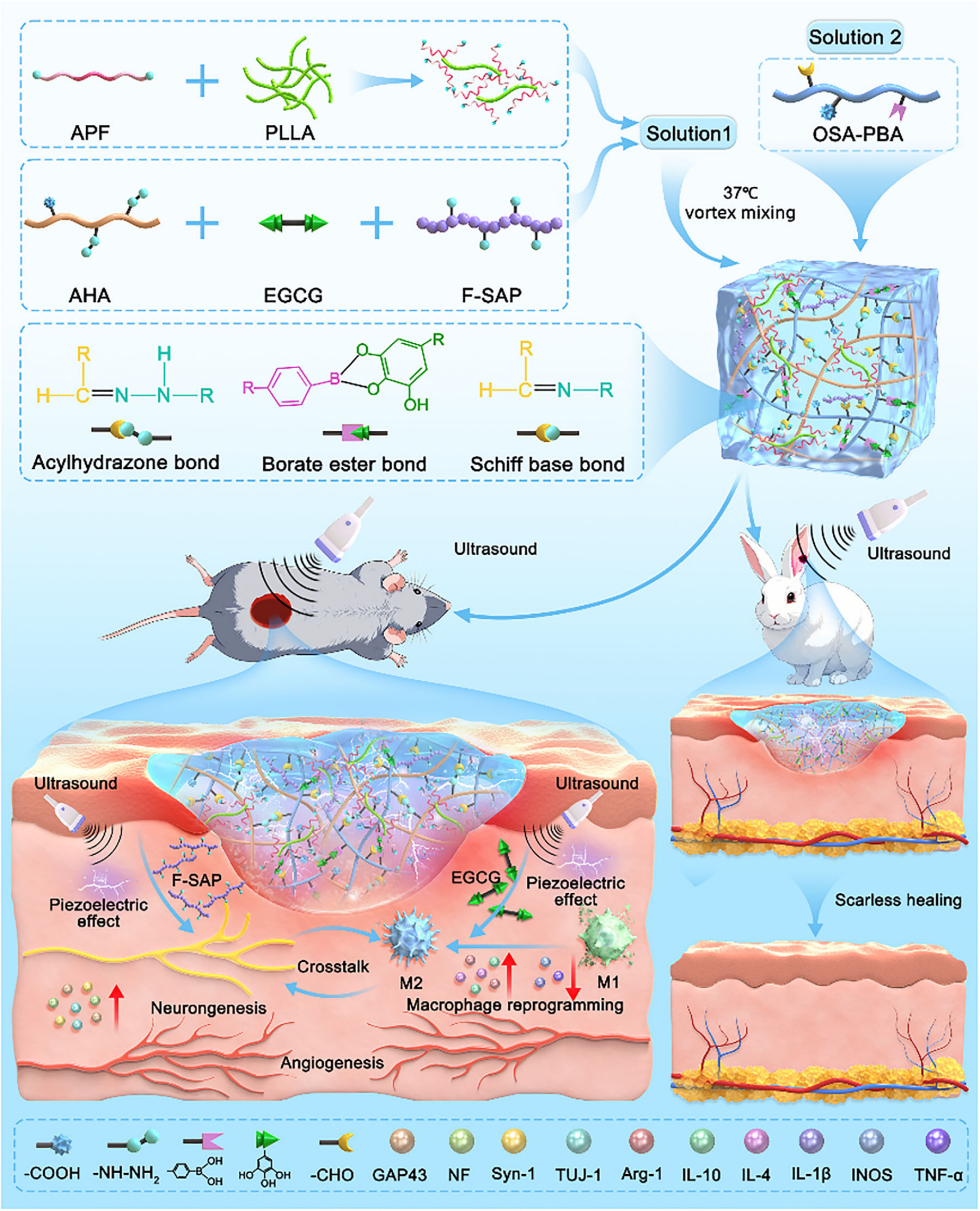
Schematic illustration of the ultrasound‐activated piezoelectric hydrogel for diabetic wound repair via neurogenesis‐macrophage reprogramming crosstalk.

To address these challenges, we designed an ultrasound‐activated piezoelectric hydrogel (AOEF@AP5) that synchronously modulates neurogenesis and macrophage polarization (Scheme 1). The hydrogel network is constructed from acylhydrazide hyaluronic acid (AHA) and phenylboronic acid–grafted oxidized sodium alginate (OSA‐PBA), which form dynamic acylhydrazone and boronate ester bonds, providing injectable, self‐healing, and tissue‐adhesive properties. EGCG introduces a second level of dynamic crosslinking via catechol–boronated interactions while imparting intrinsic antioxidant and anti‐inflammatory capacity. A functional peptide (F‐SAP) is incorporated through Schiff base bonds with OSA‐PBA, increasing crosslink density and mechanical integrity while enabling controlled release of neurotrophic cues as the hydrogel degrades and under US stimulation. Finally, biodegradable PLLA nanofibers are physically embedded and stabilized by amino‐modified Pluronic F127 (APF), which improves dispersion and forms additional dynamic imine linkages with OSA‐PBA, thereby establishing a piezoelectric phase capable of generating bioelectric signals in response to US. Benefiting from this multi‐level design, AOEF@AP5 exhibits suitable mechanical robustness, rapid self‐healing, porous architecture, high water absorption, controlled degradability, US‐activated piezoelectricity, moderate conductivity, and tissue‐adhesive yet injectable behavior. We systematically evaluated its ability to regulate neuron–macrophage crosstalk at multiple scales. In vitro, we investigated how US‐activated AOEF@AP5 modulates dorsal root ganglion (DRG) neurite outgrowth and macrophage polarization and their reciprocal interactions. In vivo, we assessed its efficacy in promoting wound closure, neurogenesis, immune remodeling, and scar attenuation in both type 2 diabetic mouse and rabbit wound models. Transcriptomic analyses of cells and wound tissues further revealed that mitochondrial functional reprogramming represents a central mechanistic axis underlying coordinated neurogenic and immunomodulatory effects. Collectively, these findings demonstrate a programmable piezoelectric hydrogel platform that couples US‐triggered bioelectric stimulation with neuroimmune modulation, offering a promising and translational strategy to overcome the complex barriers of diabetic wound healing.

## 2 Results and Discussion

### 2.1 Design, Preparation, and Characterization of AOEF@AP Hydrogels

To enable ultrasound‐activated piezoelectric regulation of neurogenesis‐macrophage crosstalk, we first constructed a dynamic covalent hydrogel network capable of integrating EGCG, F‐SAP and piezoelectric PLLA nanofibers (Figure S1). Oxidized sodium alginate grafted with phenylboronic acid (OSA‐PBA), derived from the natural polysaccharide sodium alginate (SA), served as a multifunctional crosslinker. Its abundant aldehyde groups react with hydrazide groups on acylhydrazide‐modified hyaluronic acid (AHA) to form reversible acylhydrazone bonds, providing the first dynamic crosslinking motif. In parallel, the ionic nature of SA imparts a baseline ionic conductivity to the network, which is beneficial for establishing a bioelectronic interface with regenerating nerves at the wound. The second level of dynamic crosslinking arises from the reaction between catechol groups on epigallocatechin‐3‐gallate (EGCG) and phenylboronic acid moieties on OSA‐PBA, yielding boronated ester bonds. These bonds are not only reversible but also ROS‐responsive, which adds a gated release dimension to the system. A third dynamic linkage is introduced via the self‐assembling neuroactive peptide F‐SAP. Residual primary amines on F‐SAP form Schiff base bonds with aldehyde groups on OSA‐PBA, further increasing crosslinking density and reinforcing the hydrogel network. As the matrix gradually degrades, F‐SAP is released in a sustained manner, while US stimulation can further enhance its release kinetics, providing a controllable neurotrophic input. To incorporate the piezoelectric phase, hydrophobic PLLA nanofibers were dispersed using amino‐modified Pluronic F127 (APF). Owing to its amphiphilic block copolymer structure, APF stabilizes PLLA nanofibers within the hydrophilic network. At the same time, terminal amino groups on APF can form Schiff base bonds with OSA‐PBA, modestly contributing to the dynamic crosslinking. This design allows PLLA to maintain its piezoelectric properties while being uniformly distributed in the injectable hydrogel, enabling efficient conversion of US into local electrical cues in subsequent experiments (Scheme) [70].

The key precursor polymers and peptide were structurally confirmed by ^1^H NMR, FT‐IR, and mass spectrometry (Figures S2–S4, detailed discussion is described in Note S1). Because each component plays a distinct role in neuroimmune modulation, we systematically optimize the concentrations of EGCG, F‐SAP, and PLLA (Figure S5, detailed discussion is described in Note S2). In summary, 8 mg mL^−^^1^ of EGCG, 0.5 mg mL^−^^1^ of F‐SAP, and 5 mg mL^−^^1^ of PLLA is finally chosen for our subsequent experiments, separately. Collectively, the results above confirm the successful construction of a multi‐dynamic, peptide‐ and PLLA‐integrated hydrogel network. This chemically well‐defined architecture provides the structural basis to couple US‐activated piezoelectric signaling with controlled neurotrophic peptide release, enabling subsequent programmable modulation of neurogenesis‐macrophage crosstalk in diabetic wounds.

### 2.2 Gelation Time, Rheological Property, Self‐Healing Performance, Micromorphology, Swelling, and Degradation Behavior of the Hydrogels

**FIGURE 1:**
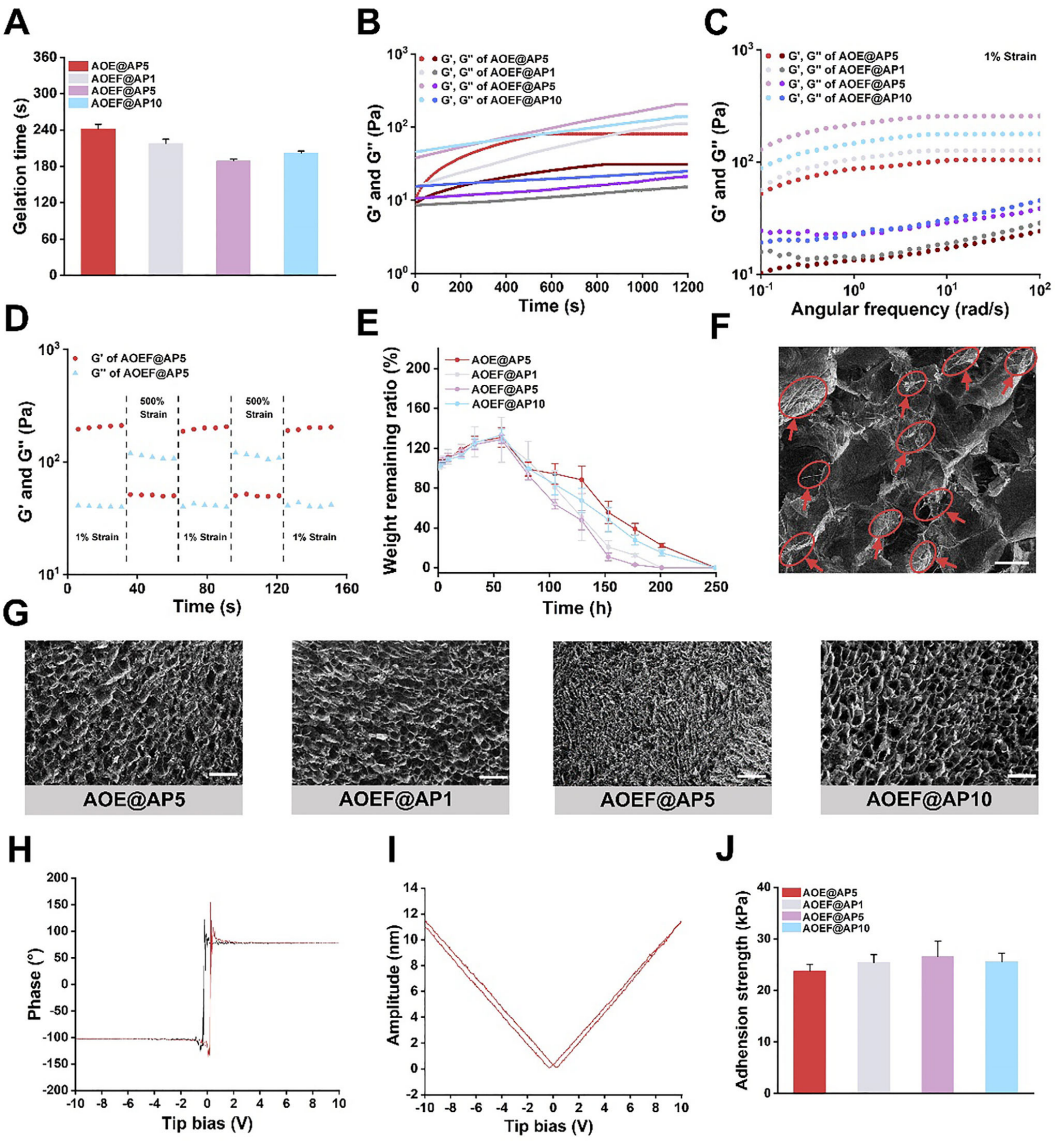
(A) Gelation time of hydrogels obtained by inversion of test tubes (n = 5). (B) Moduli of hydrogels with time at 37°C and 1% strain. (C) Frequency‐dependent rheological behavior of the hydrogels with constant strains of 1% and a frequency range of 10^−1^–10^2^ rad/s at 37°C (n = 1). (D) Self‐healing properties of the AOEF@AP5 hydrogel (n = 1). (E) Swelling and degradation behaviors of the hydrogels (n = 3). (F) Scanning electron micrographs of PLLA sticks in hydrogels, magnification: 200X, scale bar = 50 µm. (G) Microscopic morphology of hydrogel taken by scanning electron microscope, magnification: 60X, scale bar = 200 µm. (H) Phase‐voltage curve of PLLA stick (n = 1). (I) Butterfly curve of the PLLA stick (n = 1). (J) Adhesion strength of the hydrogels to pig skin (n = 6).

For an injectable dressing, gelation time and mechanical performance must match practical handling and the dynamic mechanical environment of skin [48]. In addition, self‐healing helps the hydrogel recover integrity after deformation, which is important for repeated US activation and long‐term coverage on mobile sites [49]. Gelation times were first quantified by the standard test‐tube inversion method. As shown in Figure 1A, the AOE@AP5 hydrogel (without F‐SAP) displayed the longest gelation time (241.89 s), consistent with its lower density of dynamic imine bonds. Incorporation of F‐SAP markedly accelerated gelation, and AOEF@AP1, AOEF@AP5, and AOEF@AP10 formed within 218.20, 188.85, and 202.08 s, respectively. The shorter gelation time in AOEF@AP5 compared with AOE@AP5 is attributed to the additional primary amines introduced by F‐SAP, which increase the number of Schiff‐base crosslinking sites with OSA‐PBA. The AOEF@AP5 gelled faster than AOEF@AP1, likely due to the higher APF content (PLLA and APF are used at equal mass), which further enriches amino groups and thus imine formation. In AOEF@AP10, gelation was again slightly delayed relative to AOEF@AP5, probably because excess PLLA promotes nanofiber aggregation, partially hindering network formation. Overall, all groups gelled within ∼4 min, a clinically acceptable window for in situ injection and shaping [50].

Rheological tests were then used to evaluate mechanical stability. Time‐sweep measurements showed that, for all four hydrogels, the storage modulus (G′) remained higher than the loss modulus (G″) over 1200 s at 37°C (Figure 1B), indicating a solid‐like state after gelation. G′ increased with the introduction and gradual increase of F‐SAP and APF/PLLA, reflecting higher effective crosslinking density and the reinforcing effect of hydrophobic PLLA nanofibers. Interestingly, G′ of AOEF@AP10 was lower than that of AOEF@AP5. The excessive APF may present much unreacted amino groups that do not form productive crosslinks, and excess PLLA decreases dispersion, leading to local aggregation and a lower effective modulus. Quantitatively, the maximum storage moduli (G′max) within 1200 s were 80.57 Pa (AOE@AP5), 111.00 Pa (AOEF@AP1), 203.43 Pa (AOEF@AP5), and 138.72 Pa (AOEF@AP10), respectively (Figure S6). Frequency‐sweep measurements further confirm network stability. Across the tested frequency range at 1%, 5%, and 10% strains (Figure 1C and Figure S7), G′ consistently exceeded G″ and showed only mild frequency dependence for all groups, indicating that the dynamic covalent network resists shear and maintains integrity under oscillatory loading. The similar moduli observed at 1%, 5%, and 10% strain suggest that the hydrogel network was not significantly damaged within this deformation range. The Schiff base bonds, acylhydrazone bonds, and boronate ester bonds functioned as reversible crosslinking points, allowing local bond dissociation/reformation and stress relaxation while preserving the integrity of the three‐dimensional network. Therefore, even under 10% strain, the hydrogels retained gel‐like behavior without obvious gel–sol transition or network collapse. Self‐healing was assessed by alternating low and high strain in step‐strain tests. For AOEF@AP5, at 1% strain the hydrogel exhibited a typical solid‐like response (G′ > G″). When the strain was abruptly increased to 500%, G′ dropped from ∼200 to ∼50 Pa, indicating substantial network disruption (Figure 1D). Upon returning to 1% strain, G′ rapidly recovered to ∼200 Pa with G′ again exceeding G″, demonstrating almost complete restoration of the hydrogel network. After two high‐strain cycles, no obvious fatigue in G′ was observed (Figure S8). This rapid and repeated recovery is consistent with the reversible nature of the acylhydrazone, boronate ester, and Schiff‐base bonds that can break under large deformation and re‐form once the stress is removed. Upon mechanical damage, dynamic Schiff base bonds, acylhydrazone bonds, and boronate ester bonds near the fractured interface can partially dissociate, generating reactive groups. Owing to the hydrated nature of the hydrogel and the local mobility of polymer chains, these groups can re‐contact and reform dynamic covalent crosslinks across the damaged interface. Meanwhile, hydrogen bonding, chain entanglement, and APF‐mediated interfacial interactions may further assist network reconstruction. Therefore, the self‐healing property is achieved through the synergistic reconstruction of multiple dynamic covalent and physical interactions.

Collectively, these results show that all AOEF@AP hydrogels possess rapid in situ gelation, solid‐like behavior and pronounced self‐healing. Among them, AOEF@AP5 combines the shortest gelation time, the highest storage modulus and robust self‐recovery, and was therefore selected as the optimized group for subsequent physicochemical characterization, neuroimmune regulation studies, and in vivo diabetic wound healing experiments.

An appropriate porous architecture is essential for wound dressings, as it governs exudate uptake and provides channels for gas, nutrient, and signaling‐molecule exchange between the wound bed and the hydrogel interior [51]. Therefore, the microstructures of different hydrogels were characterized in detail using scanning electron microscopy (Figure S9, SEM, detailed discussion is described in Note S3). Next, swelling and degradation behavior of all hydrogels were evaluated by tracking sample mass over time in PBS (Figures S10 and S11, detailed discussion is described in Note S3). AOEF@AP5 forms a moderately swollen, slowly degradable porous network that can absorb exudate while maintaining structural integrity over the wound‐healing window [52]. This spatiotemporally stable matrix is essential for sustaining US‐triggered piezoelectric cues and staged release of bioactive components to continuously reshape the neuroimmune microenvironment of diabetic wounds.

### 2.3 Piezoelectric Properties of PLLA Nanofibers and Adhesive/Electrical Performance of Hydrogels

**FIGURE 2:**
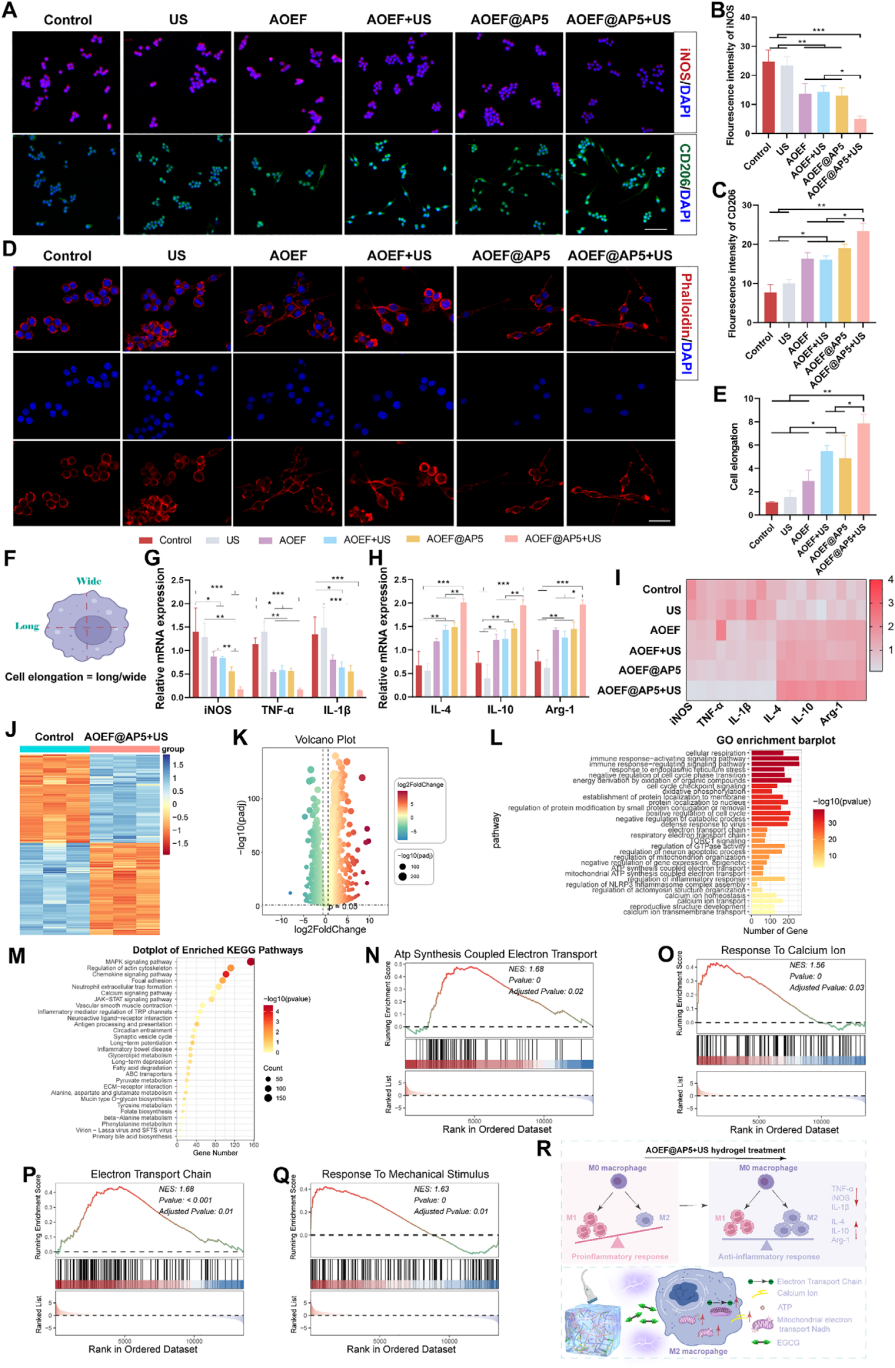
AOEF@AP5+US regulates macrophage inflammation and polarization. (A) Representative immunofluorescence photographs of iNOS (M1 macrophage marker) and CD206 (M2 macrophage marker) staining on macrophages in different groups. Scale bar: 100 µm. (B,C) Quantitative analysis of fluorescence intensity of iNOS and CD206 in different groups. (D) Representative immunofluorescence photographs of phalloidin staining on macrophages in different groups. Scale bar: 500 µm. (E) Statistical results of elongation rate of macrophage cells cultured with different groups. (F) Schematic of presenting AOEF@AP5+US‐mediated macrophage elongation. (G) qRT‐PCR assay detecting the expression levels of pro‐inflammatory cytokines iNOS, TNF‐α, and IL‐1β. (H) qRT‐PCR assay detecting the expression levels of anti‐inflammatory cytokines IL‐4, IL‐10, and Arg‐1. (I) The heatmap of expression level of mRNA iNOS, TNF‐α, IL‐1β, IL‐4, IL‐10, and Arg‐1. (J) Heatmap displaying the distribution of DEGs between control group and AOEF@AP5+US group. (K) Volcano map displaying the distribution of DEGs between control group and AOEF@AP5+US group. (L) GO enrichment analysis of DEGs between control group and AOEF@AP5+US group. (M) KEGG enrichment analysis of DEGs between control group and AOEF@AP5+US group. (N–Q) GSEA enrichment of AOEF@AP5+US group. (R) Schematic of presenting AOEF@AP5+US‐mediated M2 macrophage polarization. (n = 3, ^*^ *p* < 0.05, ^**^ *p* < 0.01, ^***^ *p* < 0.001).

To endow AOEF@AP hydrogels with ultrasound‐responsive piezoelectric signaling, we first engineered PLLA into short, highly oriented nanofibers. PLLA films obtained by electrospinning were annealed and then cut into ∼25 µm short sticks (Figure S12A), which were subsequently dispersed into the hydrogel precursor together with amino‐modified Pluronic F127 (APF) (Figure 2F). APF, a triblock copolymer with a hydrophobic core and hydrophilic termini, acts as a molecular dispersant: its terminal amines form Schiff bases with aldehydes on OSA‐PBA, anchoring APF into the dynamic covalent network, while its central hydrophobic segment associates with hydrophobic PLLA via hydrophobic interactions [53]. As a result, PLLA short sticks were stably and uniformly dispersed in the aqueous phase. In contrast, PLLA without APF formed visible aggregates within ∼18 h (Figure S12B). The surface topography and piezoelectric behavior of PLLA short sticks were characterized by AFM and PFM. AFM height images showed that annealing markedly smoothed the PLLA surface compared with non‐annealed fibers, which exhibited pronounced cone‐like protrusions (Figure S13). The root‐mean‐square roughness (Rq) decreased from 5.53 to 1.60 nm, and the average roughness (Ra) from 3.70 to 1.13 nm after annealing (Figure S14), providing a more uniform piezoelectric surface. Surface potential mapping further revealed a homogeneous negative charge distribution with an average potential of −413 mV (Figures S15 and S16). PFM measurements confirmed robust piezoelectricity. The phase‐voltage response of PLLA displayed a clear 180° phase shift upon sweeping the DC bias (Figure 1H), indicative of polarization reversal. Under an AC excitation of ±10 V, the out‐of‐plane electromechanical deformation increased progressively with bias amplitude, reached a maximum at 10 V, and then decreased during the return sweep; the response under opposite polarity followed the same trend (Figure 1I). The amplitude‐voltage curve exhibited a characteristic butterfly loop with evident hysteresis, a hallmark of piezoelectric materials [54]. Together, these data demonstrate that the annealed PLLA short sticks can efficiently transduce US‐induced mechanical stress into local electrical cues, which later serve as the upstream switch for neurogenesis‐macrophage crosstalk modulation.

After injection at the wound site, the hydrogel precursor forms a dynamic covalent network in situ while simultaneously establishing chemical anchoring to the tissue. Therefore, the adhesion properties of the hydrogel were comprehensively investigated through lap‐shear tests conducted on fresh pig skin (Figures S17 and S18, detailed discussion is described in Note S4). We next assessed bulk conductivity, as appropriate ionic conductivity can reinforce endogenous wound currents and facilitate directed migration of neutrophils, macrophages, and keratinocytes (Figure S19, detailed discussion is described in Note S4) [55]. Together, the pronounced piezoelectric response of annealed PLLA, the skin‐level conductivity, and the strong tissue adhesion of AOEF@AP5 indicate that the hydrogel can efficiently convert non‐invasive US into localized bioelectric signals at a stable wound interface. This interface is ideally suited to deliver piezoelectric neuroimmune stimulation and to engage resident nerves and macrophages in situ.

### 2.4 In Vitro Immune Modulation of the AOEF@AP5+US

Immunological dysregulation is a central barrier in diabetic wound healing [56], so we next explored whether the AOEF@AP5 hydrogel, under ultrasound activation, could actively reprogram macrophage phenotypes. AOEF@AP5+US combines three key elements, including EGCG‐mediated anti‐inflammatory signaling, PLLA‐derived piezoelectric cues, and the dynamic covalent network that stabilizes these functions in a 3D matrix (Figures S20 and S22). To evaluate macrophage polarization, RAW264.7 cells were cultured with different treatments, and phenotypic changes were assessed (Figure 2A). Immunofluorescence staining showed that AOEF@AP5+US induced the strongest M2 polarization among all groups, with markedly increased M2 marker expression compared with control and US‐only groups (Figure 2B,C). Other hydrogel groups also promoted M2 features relative to control and US, but to a lesser extent than AOEF@AP5+US. Macrophage morphology provided a complementary readout. Increased cell elongation is associated with an M2‐like state. Cytoskeletal staining revealed more spindle‐like, elongated macrophages in hydrogel groups than in controls, with AOEF@AP5+US again exhibiting the highest elongation ratio (Figure 2D–F). qRT‐PCR analysis further confirmed this immunomodulatory profile. AOEF@AP5+US significantly downregulated M1‐associated genes (iNOS, TNF‐α, IL‐1β) compared with all other groups, while the remaining hydrogel groups also showed lower M1 gene expression than control and US alone (Figure 2G,I). In parallel, AOEF@AP5+US robustly upregulated M2‐associated genes (IL‐4, IL‐10, Arg‐1), with other hydrogel groups showing intermediate increases (Figure 2H,I). These results indicate that the hydrogel matrix already provides substantial anti‐inflammatory cues (largely from EGCG and the soft ECM‐like environment), and that ultrasound is required to fully unlock the piezoelectric contribution of PLLA for maximal M2 reprogramming.

To elucidate upstream regulatory mechanisms, we performed transcriptomic profiling of macrophages treated with AOEF@AP5+US vs. control. A total of 3091 differentially expressed genes (DEGs) were identified, including 1514 upregulated and 1577 downregulated genes (Figure 2J,K). GO enrichment analysis showed that these DEGs were significantly associated with immune response‐activating signaling pathways, regulation of inflammatory responses, the respiratory electron transport chain, and calcium ion transport (Figure 2L), suggesting a tight coupling between immune signaling and mitochondrial function. KEGG analysis further highlighted enrichment of pathways such as calcium signaling, MAPK signaling, ECM‐receptor interaction, and long‐term potentiation (Figure 2M), consistent with a mechano‐ and electro‐responsive cell state. GSEA revealed that AOEF@AP5+US significantly upregulated gene sets related to ATP synthesis‐coupled electron transport, response to calcium ions, electron transport chain activity, response to mechanical stimulus, ECM‐receptor interaction, and mitochondrial electron transport from NADH to ubiquinone (Figure 2N–R and Figure S24). Overall, AOEF@AP5+US reprograms macrophages toward a pro‐regenerative M2 phenotype through coordinated regulation of calcium handling, mitochondrial electron transport, and inflammatory signaling. These data establish the immune modulation of our strategy, showing that US‐activated piezoelectric hydrogel stimulation can reshape macrophage behavior as a key component of neurogenesis‐macrophage crosstalk.

### 2.5 In Vitro Neurogenesis Modulation of the AOEF@AP5+US

**FIGURE 3:**
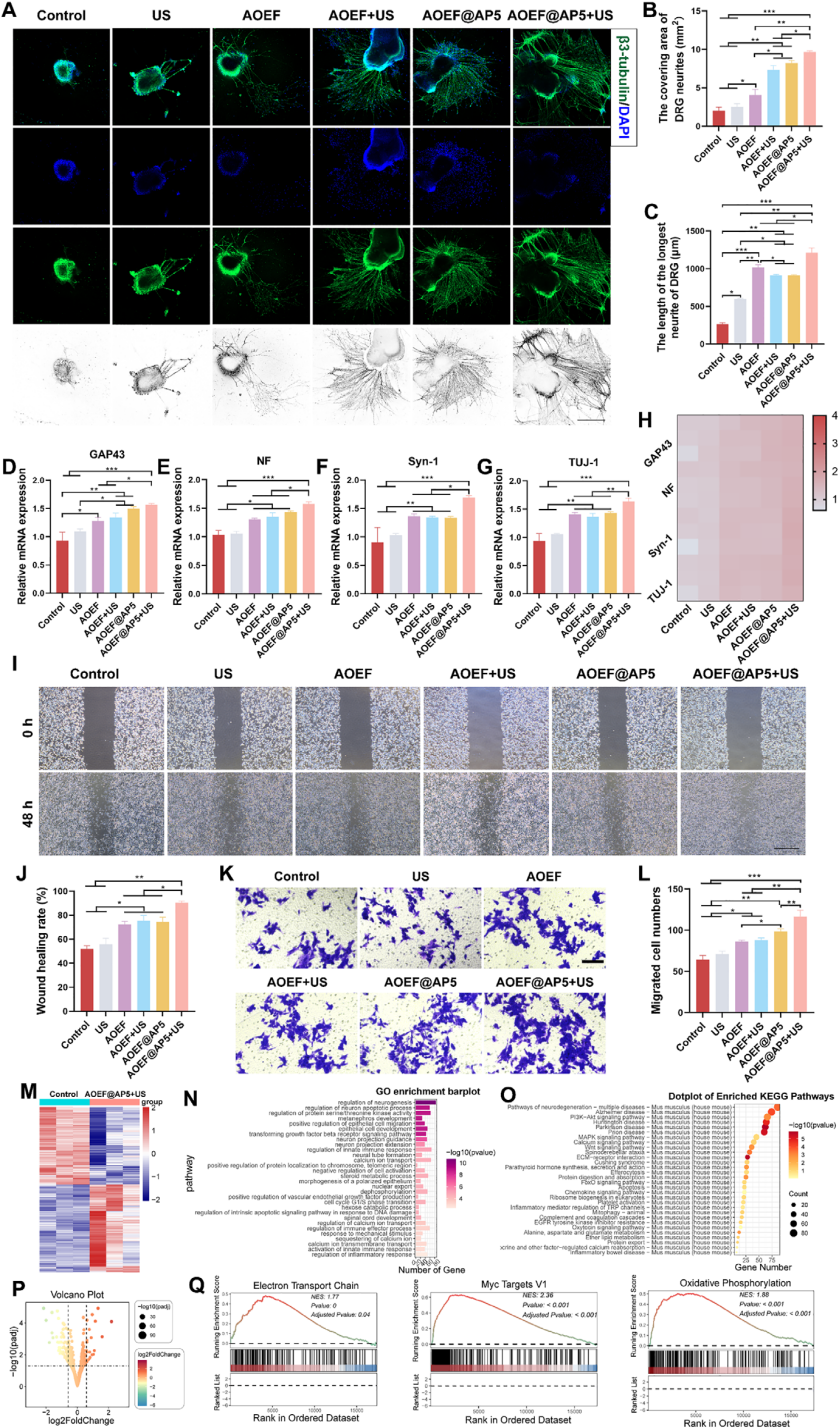
AOEF@AP5+US regulates DRG neurons regeneration and PC12 cells migration. (A) Representative immunofluorescence photographs of β3‐tubulin staining on DRG neurons in different groups. Scale bar: 500 µm. (B) Quantitative analysis of the covering area of DRG neurites in different groups. (C) Quantitative analysis of the length of the longest neurite of DRG in different groups. (D–G) qRT‐PCR assay detecting the expression levels of nerve regeneration‐associated genes GAP43, NF, Syn‐1, and TUJ‐1. (H) The heatmap of expression level of mRNA GAP43, NF, Syn‐1, and TUJ‐1. (I) Optical images of scratch assay of PC12 cells in different groups. Scale bar: 100 µm. (J) Quantitative analysis of the relative wound healing rate in different groups. (K) Optical images of transwell assay of PC12 cells in different groups. Scale bar: 200 µm. (L) Quantitative analysis of migrated cell numbers in different groups. (M) Heatmap displaying the distribution of DEGs between control group and AOEF@AP5+US group. (N) GO enrichment analysis of DEGs between control group and AOEF@AP5+US group. (O) KEGG enrichment analysis of DEGs between control group and AOEF@AP5+US group. (P) Volcano map displaying the distribution of DEGs between control group and AOEF@AP5+US group. (Q) GSEA enrichment of AOEF@AP5+US group. (n = 3, ^*^ *p* < 0.05, ^**^ *p* < 0.01, ^***^ *p* < 0.001).

Peripheral neuropathy is a hallmark barrier in diabetic wound repair [57], yet few wound dressings are designed to directly promote reinnervation within the lesion. Electrical signaling is fundamental to neuronal physiology and can enhance neurite extension and network reconstruction [58]. In AOEF@AP5+US, two neurogenic cues are deliberately coupled: F‐SAP provides a neuroactive peptide matrix, while US‐activated piezoelectric PLLA nanofibers generate local electrical signals (Figures S21 and S23). Together, they are intended to drive axonal regeneration and support the neuro‐immune axis. To assess this, we first examined the effect of AOEF@AP5+US on dorsal root ganglion (DRG) neurons. Immunofluorescence staining revealed that AOEF@AP5+US induced the most pronounced axonal outgrowth among all groups (Figure 3A). Other hydrogel groups also enhanced neurite extension compared with the control and US‐only groups, but to a lesser extent (Figure 3B,C). qRT‐PCR analysis further supported these morphological findings. AOEF@AP5+US markedly upregulated regeneration‐related genes, including GAP43, NF, Syn‐1, and TUJ‐1, compared with all other groups. The remaining hydrogel groups also showed higher expression of these genes than the control and US groups (Figure 3D–H), indicating that the dynamic AHA/OSA‐PBA/EGCG network and F‐SAP already provide a pro‐neurogenic environment, which is further amplified by ultrasound‐driven piezoelectric stimulation.

We then evaluated neuron motility. Scratch assays showed that AOEF@AP5+US most effectively accelerated neuronal migration, with a higher wound closure ratio than all other groups (Figure 3I,J). Consistently, transwell assays demonstrated that AOEF@AP5+US significantly increased the number of migrated neuronal cells (Figure 3K). Notably, AOEF@AP5 (with PLLA but without US) also promoted cell migration more than AOEF (without PLLA), suggesting that embedded PLLA contributes a baseline level of electroactive or interfacial signaling even in the absence of ultrasound (Figure 3L) [59]. These data indicate that AOEF@AP5+US enhances both neurite extension and neuronal recruitment, which are essential for reinnervation of chronic wounds.

To probe upstream regulatory mechanisms, we performed transcriptomic analysis of DRG neurons treated with AOEF@AP5+US vs. control. A total of 2405 differentially expressed genes (DEGs) were identified, including 1277 upregulated and 1128 downregulated genes (Figure 3M,P). GO enrichment analysis showed significant enrichment in biological processes related to regulation of neurogenesis, calcium ion transport, and response to mechanical stimulus (Figure 3N), consistent with a mechano‐electroresponsive neuronal state. Collectively, these results demonstrate that AOEF@AP5+US robustly enhances DRG neurite outgrowth and migration via mitochondrial metabolic reprogramming and mechano‐electrical signaling. This defines the neuronal regulatory function of our platform and, together with the macrophage data, supports a mitochondria‐centered mechanism by which US‐activated piezoelectric cues orchestrate neuroimmune reconfiguration. KEGG pathway analysis revealed that DEGs were enriched in pathways associated with neurodegeneration, calcium signaling, MAPK signaling, ECM‐receptor interaction, and long‐term potentiation (Figure 3O), all closely linked to synaptic activity and axonal plasticity. GSEA further demonstrated significant upregulation of gene sets associated with the electron transport chain, Myc targets v1, and oxidative phosphorylation in the AOEF@AP5+US group (Figure 3Q), indicating enhanced mitochondrial bioenergetics. Taken together, these results support a model in which AOEF@AP5+US delivers coordinated peptide and piezoelectric cues that boost mitochondrial oxidative phosphorylation and calcium handling in DRG neurons, thereby promoting axonal regeneration and migration. This neuronal reprogramming complements the macrophage modulation described above and forms unique platform of the neuroimmune crosstalk that AOEF@AP5+US is designed to reconstruct in diabetic wounds.

### 2.6 Neurogenesis‐Macrophage Reprogramming Crosstalk Modulated by AOEF@AP5+US

**FIGURE 4:**
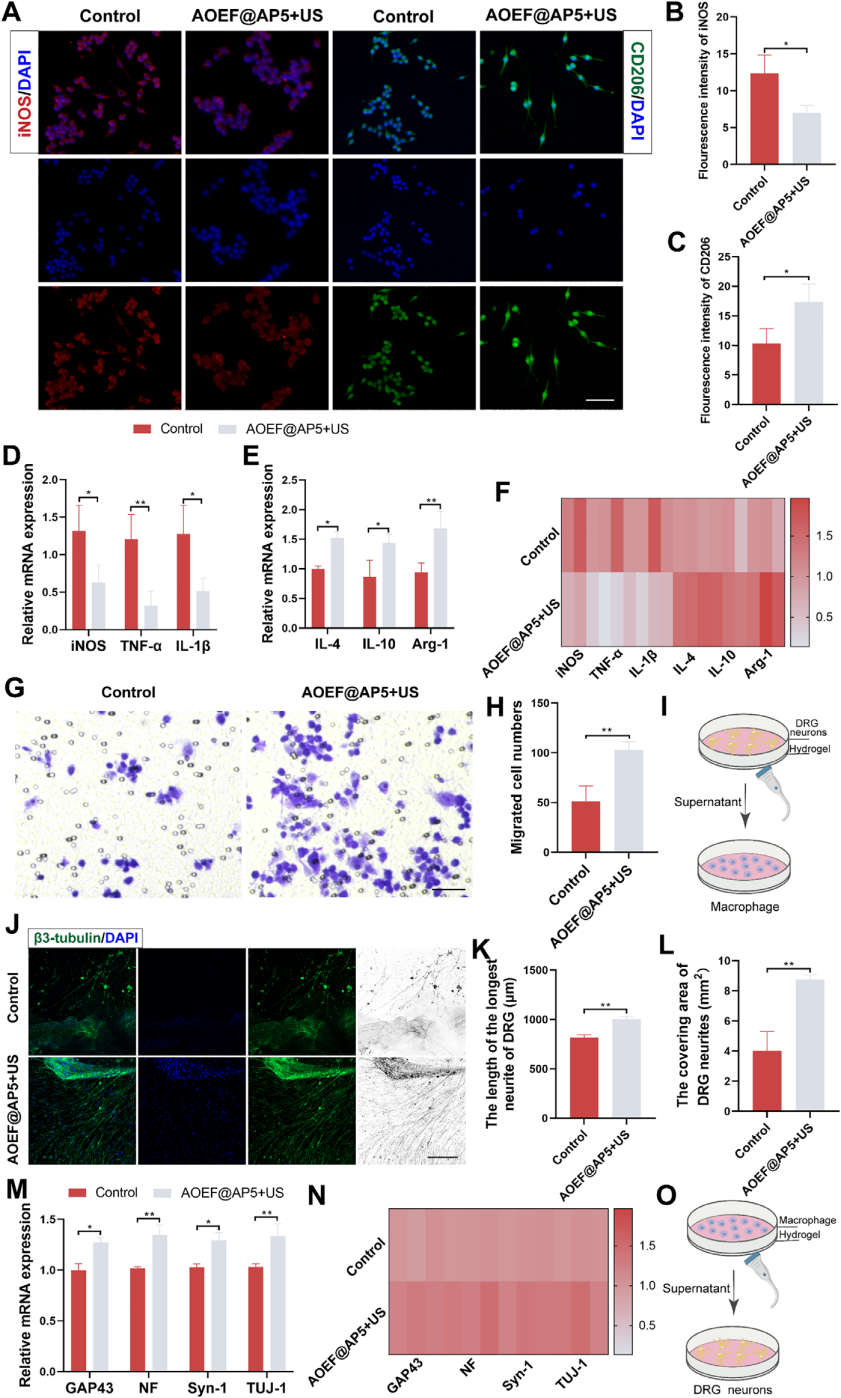
AOEF@AP5+US regulates neurogenesis‐macrophage reprogramming crosstalk. (A) Representative immunofluorescence photographs of iNOS and CD206 staining on macrophage in different groups. Scale bar: 200 µm. (B,C) Quantitative analysis of fluorescence intensity of iNOS and CD206 in different groups. (D) qRT‐PCR assay detecting the expression levels of pro‐inflammatory cytokines iNOS, TNF‐α, and IL‐1β. (E) qRT‐PCR assay detecting the expression levels of anti‐inflammatory cytokines IL‐4, IL‐10, and Arg‐1. (F) The heatmap of expression level of mRNA iNOS, TNF‐α, IL‐1β, IL‐4, IL‐10, and Arg‐1. (G) Optical images of transwell assay of macrophages in different groups. Scale bar: 200 µm. (H) Quantitative analysis of migrated cell numbers in different groups. (I) Schematic of presenting supernatants from DRG neurons treated with AOEF@AP5+US to macrophages. (J) Representative immunofluorescence photographs of β3‐tubulin staining on DRG neurons in different groups. Scale bar: 500 µm. (K) Quantitative analysis of the length of the longest neurite of DRG in different groups. (L) Quantitative analysis of the covering area of DRG neurites in different groups. (M) qRT‐PCR assay detecting the expression levels of nerve regeneration‐associated genes GAP43, NF, Syn‐1, and TUJ‐1. (N) The heatmap of expression level of mRNA GAP43, NF, Syn‐1, and TUJ‐1. (O) Schematic of presenting supernatants from macrophages treated with AOEF@AP5+US to DRG neurons. (n = 3, ^*^ *p* < 0.05, ^**^ *p* < 0.01).

**FIGURE 5:**
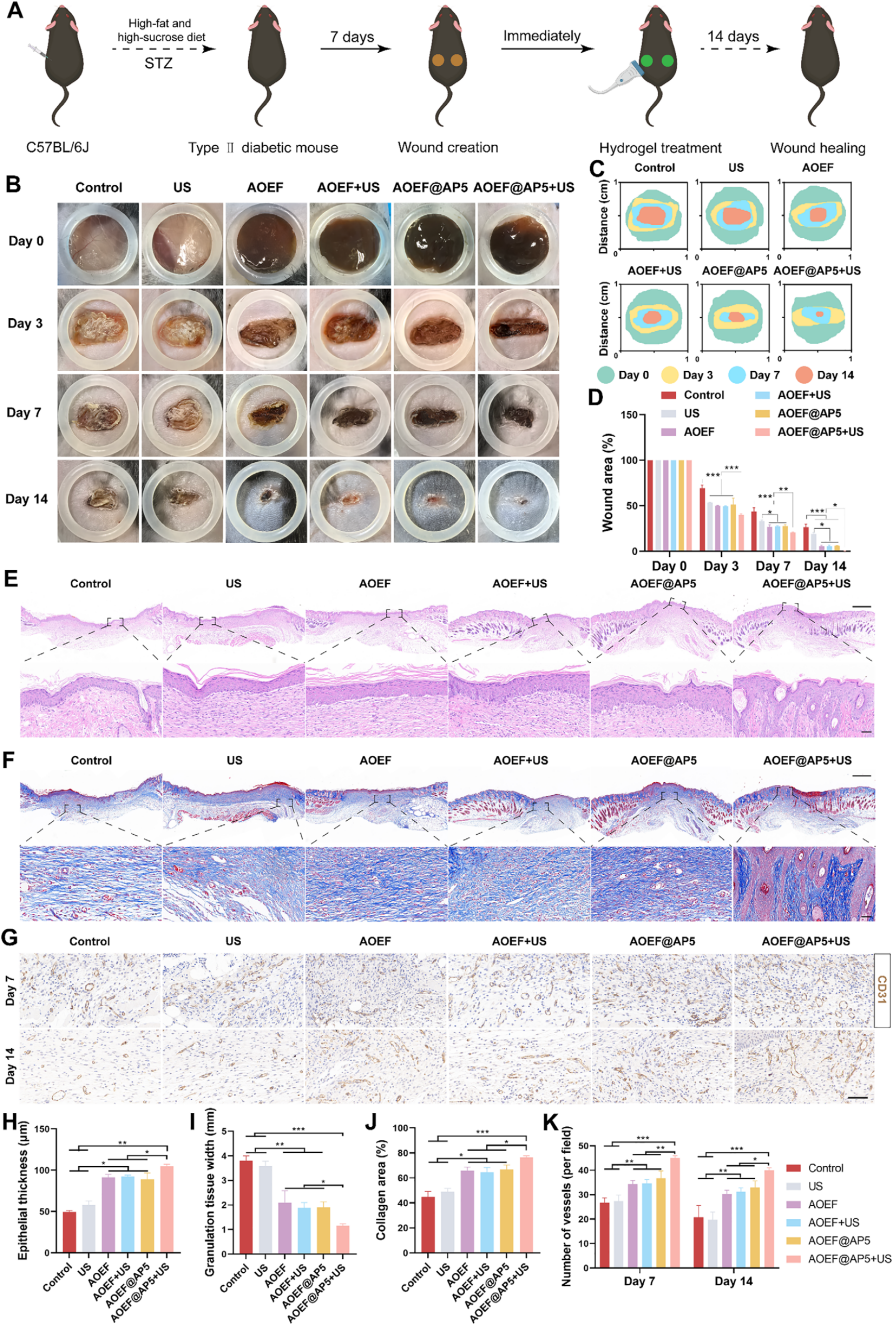
AOEF@AP5+US facilitated diabetic wound healing in vivo. (A) Schematic of presenting AOEF@AP5+US‐mediated diabetic wound healing. (B) Wound pictures of diabetic mice at different time points after different treatments. (C) Diagram of the wound healing process. (D) Quantitative analysis of the relative wound area at different times. (E) Representative H&E staining images of wounds on day 14. Scale bar: 100 µm. (F) Representative Masson's trichrome staining images of wounds on day 14. Scale bar: 100 µm. (G) Representative immunohistochemical staining of CD31 images of wounds on day 7 and day 14. Scale bar: 200 µm. (H) Quantitative analysis of epidermal thickness on day 14. (I) Quantitative analysis of granulation tissue width on day 14. (J) Quantitative analysis of the relative collagen area on day 14. (K) Quantitative analysis of the number of vessels on day 7 and day 14. (n = 5, ^*^ *p* < 0.05, ^**^ *p* < 0.01, ^***^ *p* < 0.001).

Our previous work has shown that neurogenesis‐macrophage crosstalk is a key amplifier of tissue repair: peripheral nerve‐derived CSF1 drives BMP2 expression in pro‐repair macrophages, which in turn supports nerve regeneration and accelerates wound healing [16, 18]. This establishes a positive feedback loop between nerves and macrophages. In diabetic wounds, concurrent neuropathy and macrophage dysfunction severely disrupt this axis and constitute a major barrier to healing [60, 61]. AOEF@AP5 + US was therefore designed not only to act on neurons and macrophages individually, but also to re‐engage their bidirectional communication. To test whether AOEF@AP5 + US can enhance this crosstalk, we performed conditioned‐medium experiments in both directions (Figures 4I and 5O). First, macrophages were treated with supernatants collected from DRG neurons preconditioned with different hydrogels. Immunofluorescence staining showed that supernatants from AOEF@AP5+US‐treated DRG cultures markedly reduced M1 polarization and enhanced M2 polarization compared with the control (Figure 4A–C). qRT‐PCR confirmed this shift at the transcriptional level, where iNOS, TNF‐α, and IL‐1β were significantly downregulated, whereas IL‐4, IL‐10, and Arg‐1 were robustly upregulated in macrophages exposed to AOEF@AP5+US–DRG supernatants (Figure 4D–F). Transwell assays further indicated that these supernatants enhanced macrophage recruitment relative to the control (Figure 4G,H), suggesting that AOEF@AP5 + US reprograms DRG neurons to secrete factors that actively attract and polarize macrophages toward a pro‐regenerative M2 state.

We then studied whether macrophages modulated by AOEF@AP5 + US, in turn, can support neuronal regeneration. DRG neurons were cultured with supernatants from macrophages pretreated with the various groups. Conditioned media from AOEF@AP5+US‐treated macrophages significantly promoted axonal outgrowth, as reflected by increased axonal length and expanded neurite area relative to the control (Figure 4J–L). Consistently, qRT‐PCR revealed higher expression of neuroregeneration‐related genes GAP43, NF, Syn‐1, and TUJ‐1 in DRG neurons exposed to AOEF@AP5+US‐macrophage supernatants compared with the control group (Figure 4M,N). These data indicate that AOEF@AP5 + US not only directly benefits DRG neurons and macrophages, but also reshapes their secretomes to reinforce a reciprocal, pro‐healing communication.

RNA‐seq provided further insight into the upstream mechanisms driving this coordinated response. When DRG neurons or macrophages were stimulated with AOEF@AP5 + US, differentially expressed genes were enriched in pathways related to calcium signaling, mitochondrial electron transport, and ATP synthesis. These signatures suggest that piezoelectric signals, F‐SAP and EGCG‐mediated biochemical cues converge on mitochondrial and calcium‐dependent programs, enhancing cellular activation and reprogramming. In both cell types, AOEF@AP5 + US upregulated mitochondrial and Ca^2^^+^‐associated pathways, which is consistent with the phenotypic shifts described above [62]. Taken together, the supernatant transfer experiments and transcriptomic analyses show that AOEF@AP5+US does not act on neurons or macrophages in isolation, but amplifies a bidirectional, mitochondria‐ and calcium‐dependent communication between the two cell types. This positive feedback loop at the neuroimmune interface represents the core mechanistic axis through which our US‐activated piezoelectric hydrogel may drive coordinated tissue repair.

### 2.7 In Vivo Evaluation of Diabetic Wound Healing and Angiogenesis

Effective diabetic wound dressings must not only be cytocompatibility, but also actively counteract the hostile microenvironment and cellular dysfunction that sustain chronic non‐healing wounds [51, 63, 64]. To assess the therapeutic performance of AOEF@AP5 + US in vivo, a type II diabetic (T2DM) mouse model was established and circular full‐thickness dorsal skin defects were created (Figure 5A). Mice were randomly assigned to six groups, including Control, US, AOEF, AOEF+US, AOEF@AP5, and AOEF@AP5+US (Figure 5B,C). Serial wound imaging showed that AOEF@AP5+US markedly accelerated wound closure compared with all other groups. By day 3, the AOEF@AP5+US group already exhibited a smaller residual wound area, and this advantage became more pronounced on days 7 and 14 (Figure 5D). The other hydrogel‐treated groups and the US‐only group also improved closure relative to the untreated control, but to a lesser extent than AOEF@AP5+US, underscoring the importance of combining piezoelectric stimulation with neuroactive and immunomodulatory cues.

Histological analyses further confirmed the superior repair quality in the AOEF@AP5+US group. H&E staining on day 14 revealed more complete re‐epithelialization and thicker neo‐epidermis in the AOEF@AP5+US group compared with all other groups, while the remaining hydrogel groups also showed increased epidermal thickness relative to the Control and US groups (Figure 5E,H). In parallel, the distance between wound edges (granulation gap) was shortest in the AOEF@AP5+US group, indicating more advanced tissue bridging; the other hydrogel groups again outperformed the Control and US groups (Figure 5I). Masson's trichrome staining demonstrated that AOEF@AP5+US also promoted more robust collagen remodeling. The collagen‐positive area fraction in the AOEF@AP5+US group was significantly higher than in the other treatment groups, which themselves exceeded the Control and US groups (Figure 5F,J). The collagen fibers in AOEF@AP5+US‐treated wounds appeared denser and more organized, suggesting improved matrix deposition and maturation.

**FIGURE 6:**
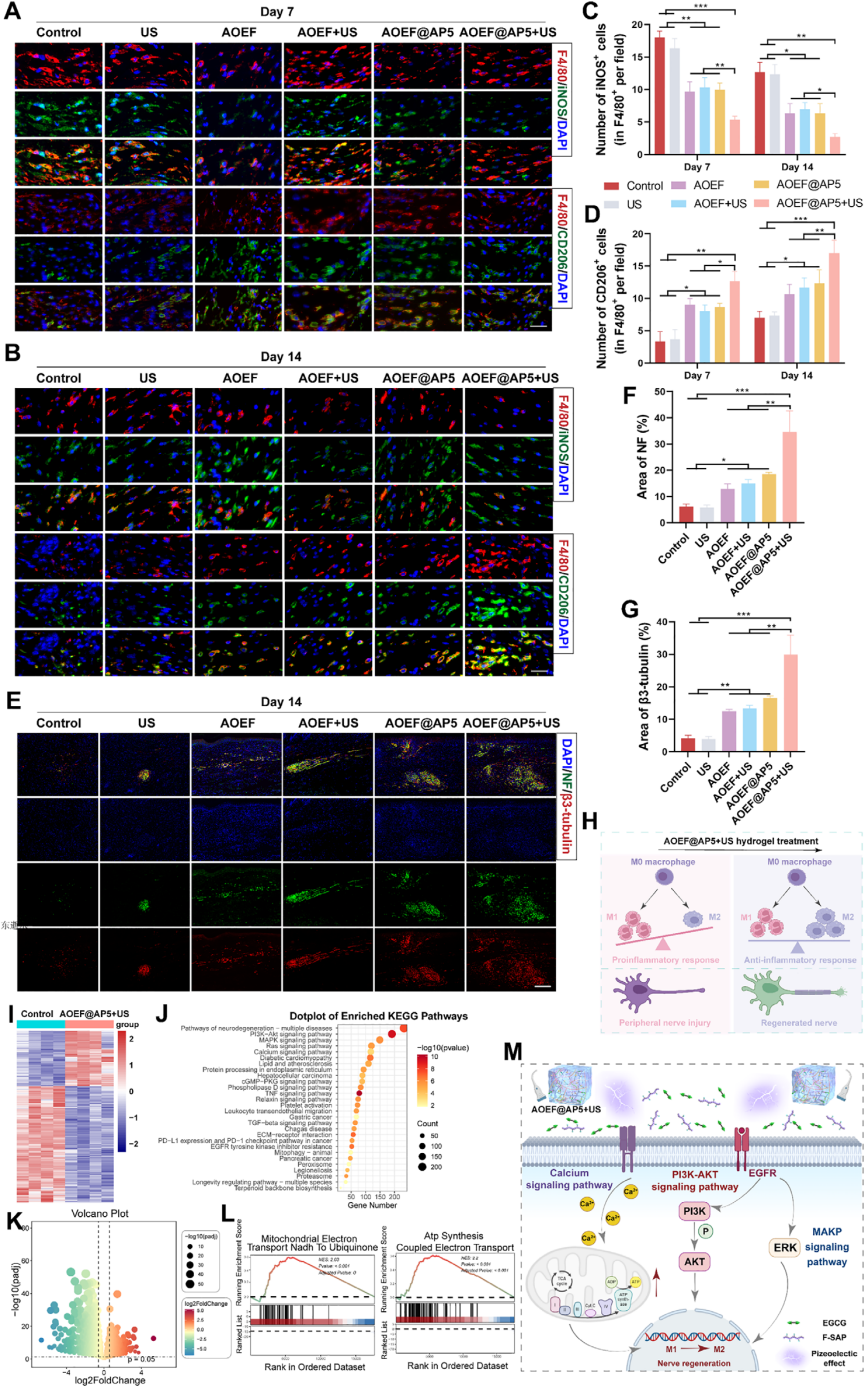
AOEF@AP5+US induces neurogenesis and macrophages reprogramming and transcriptomic analysis in vivo. (A) Representative immunofluorescence staining of F4/80, iNOS and CD206 images of wounds on day 7. Scale bar: 100 µm. (B) Representative immunofluorescence staining of F4/80, iNOS and CD206 images of wounds on day 14. Scale bar: 100 µm. (C) Quantitative analysis of the number of iNOS^+^ positive and F4/80^+^ positive cells on day 7 and day 14. (D) Quantitative analysis of the number of CD206^+^ positive and F4/80^+^ positive cells on day 7 and day 14. (E) Representative immunofluorescence staining of NF and β3‐tubulin images of wounds on day 14. Scale bar: 100 µm. (F) Quantitative analysis of the relative area of NF on day 14. (G) Quantitative analysis of the relative area of β3‐tubulin on day 14. (H) Schematic of presenting AOEF@AP5+US‐mediated regulation of neuro‐immune. (I) Heatmap displaying the distribution of DEGs between control group and AOEF@AP5+US group. (J) KEGG enrichment analysis of DEGs between control group and AOEF@AP5+US group. (K) Volcano map displaying the distribution of DEGs between control group and AOEF@AP5+US group. (L) GSEA enrichment of AOEF@AP5+US group. (M) Schematic of presenting AOEF@AP5+US‐mediated mechanism of wound repair. (n = 3, ^*^ *p* < 0.05, ^**^ *p* < 0.01, ^***^ *p* < 0.001).

Neovascularization was evaluated by immunohistochemical staining for CD31 (Figure 6G). The AOEF@AP5+US group exhibited the highest microvessel density, whereas the other hydrogel groups showed intermediate increases compared with Control and US groups (Figure 5K). This enhancement in angiogenesis is consistent with the in vitro findings that AOEF@AP5+US promotes both macrophage M2 polarization and DRG neurite outgrowth, together creating a pro‐regenerative, pro‐angiogenic microenvironment. To examine systemic biosafety, major organs (heart, liver, spleen, lungs, and kidneys) were collected after 14 days of treatment and analyzed by H&E staining. No obvious necrosis, hemorrhage, hyperplasia, or other pathological alterations were observed in any group (Figure S25), indicating good in vivo biocompatibility of the hydrogel system and the US regimen used. Thus, in a T2DM mouse model, AOEF@AP5+US translates it's in vitro neuroimmune activities into accelerated wound closure, enhanced re‐epithelialization, mature collagen remodeling, and robust angiogenesis. These in vivo outcomes indicate that US‐triggered piezoelectric stimulation of the neuroimmune axis can effectively overcome the chronic inflammatory and ischemic barriers characteristic of diabetic wounds.

### 2.8 In Vivo Modulation of Neurogenesis‐Macrophage Reprogramming and Mechanistic Insights Into Wound Repair

The above data demonstrate that AOEF@AP5+US markedly accelerates closure of diabetic wounds. Then, we explored how this platform reshapes the neuro‐immune microenvironment in vivo. To this end, neurogenesis and macrophage polarization were evaluated in T2DM wound tissues on days 7 and 14 by immunofluorescence staining (Figure 6A,B). On day 7, AOEF@AP5+US already induced a pronounced shift in macrophage phenotype. Compared with all other groups, AOEF@AP5+US reduced the proportion of iNOS^+^ M1 macrophages and increased CD206^+^ M2 macrophages within the wound bed, whereas the other hydrogel groups also showed a moderate improvement over Control and US alone (Figure 6C). By day 14, this pattern was further consolidated, where AOEF@AP5+US maintained the lowest M1 burden and the highest M2 level among all groups, while the remaining hydrogel‐treated wounds still outperformed Control and US groups (Figure 6D). These results indicate that AOEF@AP5+US sustains pro‐regenerative macrophage reprogramming in vivo rather than providing only a transient effect. We then assessed reinnervation in the same wounds. Immunofluorescence staining for NF and β3‐tubulin on day 14 revealed that AOEF@AP5+US significantly enhanced axonal regeneration, with a larger NF^+^/β3‐tubulin^+^ area compared with Control, US, AOEF, AOEF+US, and AOEF@AP5 (Figure 6E–G). The other hydrogel groups also promoted axonal regrowth relative to Control and US alone, but to a lesser extent than AOEF@AP5+US. Together, these in vivo data support that AOEF@AP5+US concomitantly drives M2 polarization and nerve regeneration, establishing a positive feedback loop of neurogenesis‐macrophage crosstalk that reinforces tissue repair (Figure 6H).

To elucidate the underlying mechanisms at the tissue level, RNA‐seq was performed on wound tissues from AOEF@AP5+US and Control groups. A total of 1156 differentially expressed genes (DEGs) were identified, including 373 upregulated and 783 downregulated genes in the AOEF@AP5+US group (Figure 6I,K). Gene Ontology analysis showed that these DEGs were significantly enriched in processes related to skin development, electron transport chain activity, and response to mechanical stimulus (Figure S26A), consistent with a mechanically gated, metabolism‐linked repair program. KEGG analysis further revealed enrichment in pathways of neurodegeneration, calcium signaling, and MAPK signaling (Figure 6J), aligning with our in vitro observations in DRG neurons and macrophages. GSEA provided additional insight into the metabolic and signaling programs engaged in AOEF@AP5+US‐treated wounds. The AOEF@AP5+US group exhibited significant upregulation of mitochondrial electron transport from NADH to ubiquinone, ATP synthesis‐coupled electron transport, and the respiratory electron transport chain, as well as pathways related to calcium‐independent cell‐cell adhesion (Figure 6L and Figure S26B,C). These signatures point to a coordinated enhancement of mitochondrial oxidative phosphorylation and cellular communication within the regenerating tissue.

Integrating these datasets, we propose the following working model (Figure 6M). Previous literature has shown that electrical signal transmission can enhance oxidative respiration capacity by regulating mitochondrial function, thereby influencing changes in cell phenotypes [65]. Based on this key point, our research developed hydrogels and further applied them to cells and tissues. Through sequencing, similar conclusions were discovered. Under US stimulation, AOEF@AP5+US converts acoustic energy into local piezoelectric signals, which, together with EGCG‐mediated anti‐inflammatory effects and F‐SAP‐driven neurotrophic cues, activate calcium channels and mechanosensitive pathways in wound‐resident cells. The resulting calcium influx and bioelectric stimulation upregulate mitochondrial oxidative respiration and engage PI3K/AKT and MAPK signaling cascades, thereby reprogramming transcription toward pro‐healing phenotypes. In macrophages, this favors M2 polarization and resolution of inflammation; in neurons, it supports axonal regeneration and neuropeptide production. The restored neurogenesis‐macrophage crosstalk further amplifies these effects, closing the loop of neuroimmune regulation. These findings support a unifying mechanism in which US‐activated piezoelectric cues rewire neurogenesis‐macrophage crosstalk through mitochondrial functional reprogramming in the diabetic wound bed.

### 2.9 In Vivo Evaluation of Scar‐Modulating Effects in a Rabbit Hypertrophic Scar Model

**FIGURE 7:**
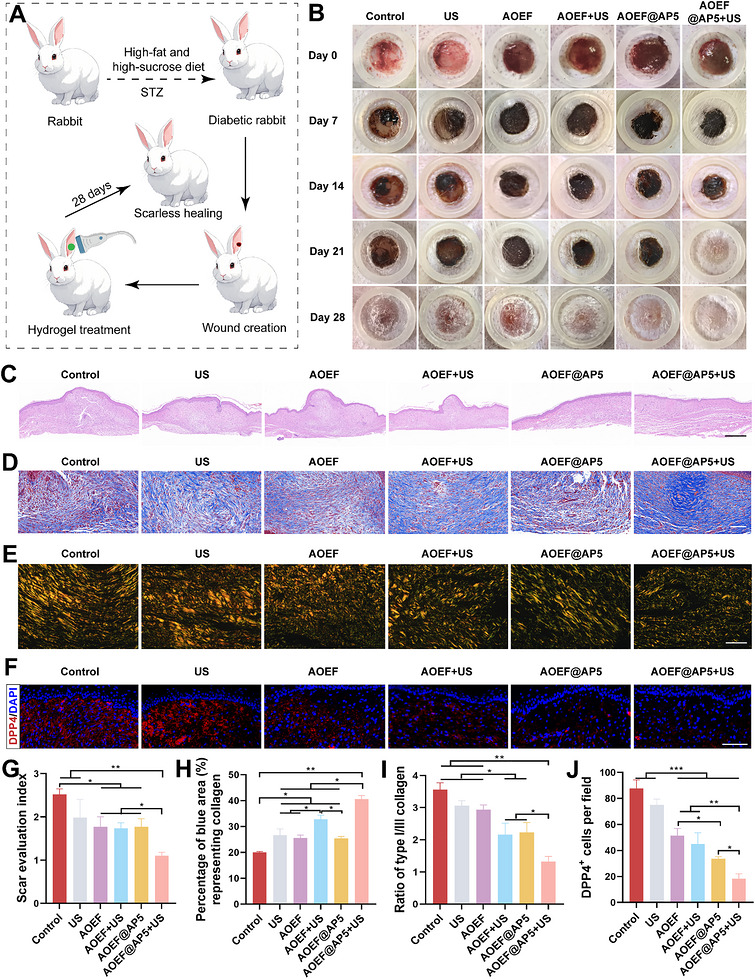
The AOEF@AP5+US inhibited scar formation. (A) Schematic of presenting AOEF@AP5+US‐mediated healing on diabetic wound of rabbits. (B) Wound pictures of diabetic rabbits at different time points after different treatments. (C) Representative H&E staining images of wounds on day 28. Scale bar: 100 µm. (D) Representative Masson's Trichrome staining images of wounds on day 28. Scale bar: 200 µm. (E) Representative Sirius Red staining images of wounds on day 28. Scale bar: 200 µm. (F) Representative immunofluorescence staining of DPP4 images of wounds on day 28. Scale bar: 200 µm. (G) Quantitative analysis of the scar evaluation index of wound tissues. (H) Percentages of collagens in skin wound tissues. (I) Quantitative analysis of type I/III collagen ratio of skin wound tissues. (J) Quantitative analysis of the number of DPP4^+^ cells per field on day 14. (n = 3, ^*^ *p* < 0.05, ^**^ *p* < 0.01, ^***^ *p* < 0.001).

We next examined whether AOEF@AP5+US could also modulate post‐injury remodeling and limit scar formation in a more clinically relevant setting. A rabbit ear hypertrophic scar model [66], which closely mimics human dermal scarring, was established and full‐thickness wounds were treated with Control, US, AOEF, AOEF+US, AOEF@AP5, or AOEF@AP5+US (Figure 7A). Macroscopically, wounds in the AOEF+US, AOEF@AP5, and AOEF@AP5+US groups closed more rapidly than those in the Control and US groups (Figure 7B). By day 28, Control, US, and AOEF groups developed obvious red, elevated scars, whereas AOEF+US, AOEF@AP5, and especially AOEF@AP5+US formed flatter, paler tissue. Among all treatments, AOEF@AP5+US produced wound surfaces that most closely resembled normal skin, suggesting a pronounced anti‐scar effect. Histological analysis further supported this observation. H&E staining revealed typical hypertrophic features in Control wounds, including thickened, uneven epidermis and disorganized dermis (Figure 7C). In contrast, AOEF@AP5+US treatment yielded a thinner, more uniform epidermis and a more normalized dermal architecture, which was reflected by a significantly lower scar elevation index compared with other groups (Figure 7G). Collagen remodeling was evaluated by Masson's trichrome and Sirius Red staining. In the AOEF@AP5+US group, collagen fibers were more finely arranged and closely resembled that of normal tissue, while also covering a larger, more homogeneous area (Figure 7D,H). Quantitative analysis showed that the type I/III collagen ratio in AOEF@AP5+US‐treated skin was significantly reduced compared with all other groups (Figure 7E,I), indicating a shift toward a more regenerative, less fibrotic matrix composition.

To further assess scar fibroblast activity, we quantified DPP4^+^ cells, which are highly expressed in scar‐associated fibroblasts and correlate with scar severity [67]. DPP4^+^ cell numbers did not differ significantly between the Control and US groups. In contrast, all hydrogel‐treated groups exhibited reduced DPP4^+^ cell counts, with AOEF@AP5+US showing the lowest level among all treatments (Figure 7F,J). No significant differences were observed between AOEF and AOEF+US, or between AOEF+US and AOEF@AP5, suggesting that US and PLLA each modestly influence fibroblast behavior, while the full AOEF@AP5+US combination exerts the strongest anti‐scar effect. Mechanistically, mechanical tension generated during rabbit ear wound contraction and excessive collagen deposition likely deforms the embedded PLLA nanofibers, inducing piezoelectric signals even in the absence of external US [68]. These endogenous or US‐amplified electrical cues, together with EGCG, F‐SAP, and the dynamic covalent network, are expected to modulate fibroblast activation and matrix production, reducing their propensity to adopt a scar‐forming phenotype. According to previous literature, DPP4 is a key gene for scar formation. Inhibiting the expression of DPP4 can effectively inhibit the transformation of myofibroblasts [69]. In this study, the AOEF@AP5+US hydrogel can effectively inhibit the expression of DPP4, inhibiting the transformation into myofibroblasts, and ultimately promoting scarless wound healing. Overall, AOEF@AP5+US not only accelerates closure of diabetic wounds but also normalizes collagen architecture, reduces profibrotic DPP4^+^ fibroblasts, and attenuates hypertrophic scar formation in a rabbit model. These results extend the concept of US‐activated piezoelectric neuroimmune modulation from promoting repair to improving the quality of regeneration, underscoring the translational potential of this strategy for functional, scar‐limited skin healing.

## 3 Conclusion

In this work, we design an ultrasound‐activated piezoelectric hydrogel platform (AOEF@AP5+US) that reprograms neurogenesis‐macrophage crosstalk as an integrated strategy to overcome the intertwined barriers of chronic inflammation, peripheral neuropathy, and impaired regeneration in diabetic wounds. By embedding piezoelectric PLLA nanofibers and a neuroactive self‐assembling peptide (F‐SAP) within a dynamic covalent AHA/OSA‐PBA/EGCG network, the hydrogel converts noninvasive US into spatially confined electrical and biochemical cues while maintaining injectability, self‐healing, tissue adhesion, and ROS/degradation responsiveness. In vitro, AOEF@AP5+US simultaneously promoted M2‐biased macrophage polarization and dorsal root ganglion (DRG) axonal outgrowth, and supernatant‐exchange experiments confirmed a bidirectional, pro‐regenerative dialogue between macrophages and neurons. RNA‐seq of both cell types revealed convergent engagement of mitochondrial oxidative phosphorylation, calcium signaling, mechanotransduction, and ECM‐receptor pathways. This supports a mechanism in which US‐driven piezoelectric signaling, together with EGCG and F‐SAP, manipulates mitochondrial and transcriptional programs to stabilize a pro‐healing neuroimmune state. In type 2 diabetic mouse wounds, AOEF@AP5+US accelerated wound closure, enhanced re‐epithelialization, collagen remodeling, angiogenesis, and reinnervation, and shifted the in situ macrophage landscape toward an M2‐dominant phenotype. In a diabetic rabbit ear model, AOEF@AP5+US further reduced hypertrophic scar formation, normalized collagen architecture and type I/III ratios, and decreased DPP4^+^ scar‐associated fibroblasts, indicating that the same neuroelectric and immunomodulatory principles can be extended to guide more scar‐like‐normal repair. This work offers a conceptually and technically versatile platform that may be adapted to other chronic, neuroimmune‐driven pathologies in regenerative medicine.

## 4 Materials and Methods

### 4.1 Materials

Hyaluronic acid (HA, molecular weight: 200 000–400 000) was purchased from Huaxi Biotechnology Co., LTD. Dihydrazide adipate (ADH) was purchased from Shanghai Bidd Pharmaceutical Technology Co., LTD. Sodium alginate (SA, stickiness 180–220 mPa s), sodium periodate (NaIO_4_), Pluronic F127 (PF127), and N, N‐carbonyldiimidazole (CDI) were purchased from Sigma–Aldrich. 3‐aminophenylboric acid, 1‐(3‐Dimethylaminopropyl)‐3‐ethyl carbon diimide hydrochloride (EDC∙HCl) and N‐hydroxysuccinimide (NHS) were purchased from Macklin. Epigallocatechin‐3‐gallate (EGCG) was purchased from MedChemExpress LLC. 2‐(N‐Morpholino) ethanesulfonic acid was purchased from J&K Scientific.

### 4.2 Synthesis of Hydrazide Hyaluronic Acid (AHA)

3 g hyaluronic acid was dissolved in 200 mL deionized water, after which 8.8 g ADH was added to the solution above. Then, 2.86 g EDC∙HCl and 1.71 g NHS were dissolved in 100 mL of deionized water and added to the above reaction. Adjust the pH of the solution to 6.8 with 1 mol/L HCl. After stirring the solution at room temperature for 12 h, the product was dialyzed with deionized water for 4 days. After adding 5 wt.% NaCl, the obtained solution was precipitated in pre‐cooled ethanol. The precipitate was dissolved in deionized water again and dialysis was performed for 4 days to remove the salt. Finally, the final AHA product was obtained through freeze‐drying.

### 4.3 Synthesis of Sodium Alginate Oxide Graft Phenylboric Acid (OSA‐PBA)

1 g sodium alginate (SA) was dissolved in 250 mL MES (0.1 m) buffer solution, the pH of which was adjusted to 5.5 using 0.1 m HCl. Then, 700 mg 1‐(3‐Dimethylaminopropyl)‐3‐ethyl carbon diimide hydrochloride (EDC∙HCl) and 100 mg N‐hydroxysuccinimide (NHS) were added to the SA solution above. 300 mg 3‐aminophenylboric acid (PBA) was added into the mixed solution and reacted at room temperature for 12 h. The resulting reaction solution was dialyzed for 3 days (MWCO:3500), during which the pH of the solution was maintained at 5.5, and the product was obtained after freeze‐drying. Next, the obtained SA‐PBA (1 g, 5.05 mm) was dissolved in 100 mL of deionized water. NaIO_4_ (1.08 g) was then dissolved in 20 mL of deionized water and added drop by drop to the solution above. The reaction solution was stirred at 30°C for 2 h in a dark environment, and finally excessive ethylene glycol was added to stop the oxidation reaction. The above solution was fully dialyzed in neutral deionized water (MWCO:3500) and freeze‐dried to obtain pure OSA‐PBA product.

### 4.4 Synthesis of F‐SAP Polypeptide

The functionalized peptide RADA16‐IKVAV [Ac‐(Arg‐Ala‐Asp‐Ala)‐Arg‐Ile‐Lys‐Val‐Ala‐Val] powder, termed F‐SAP, was custom‐synthesized by QYAOBIO (Shanghai, China) with a purity above 95%.

### 4.5 Synthesis of Piezoelectric PLLA Nanofiber

Piezoelectric PLLA nanofibers were prepared by electrospinning. 1 g PLLA was dissolved in 20 mL of chloroform overnight. A G22 needle was used to spin at a flow rate of 2 mL/h at 14 kV and collected on a cylinder at 4000 rpm to obtain aligned nanofiber pads with humidity control of 25%–45%. The resulting PLLA was annealed overnight at 100°C and slowly cooled to room temperature, then the annealing process is repeated at 160°C. Finally, the obtained oriented PLLA fiber membranes were OCT‐embedded and freeze‐cut into short fiber clusters with a length of 15–25 µm, and frozen at −20°C for spare use.

### 4.6 Synthesis of Aminated PF‐127 (APF)

The method for the preparation of aminated PF127 (APF) has been reported to be synthesized by amination of the two terminal hydroxyl groups of PF127 as follows. First, 1 mmol of PF127 (12.7 g) was placed in a 500 mL three‐necked round‐bottomed flask and dried under vacuum in an oil bath at 80°C for 12 h. The completely dried PF127 was dissolved in 20 mL of anhydrous dichloromethane in an ice‐water bath. Subsequently, 3.24 g of CDI was dissolved in 25 mL of anhydrous dichloromethane and added dropwise under nitrogen flow to a three‐necked round‐bottomed flask. After reacting for 12 h at room temperature, 10 mL of ethylenediamine was added and the reaction was continued for 12 h. Next, 300 mL of deionized water was added at once and shaken well, and then extracted with dichloromethane. The solution obtained from the extraction was purified by washing with saturated brine for three times. Finally, the washed solution was precipitated in cold ether, and the purified APF was evaporated and dried.

### 4.7 Preparation of AOEF@AP Hydrogels

First, for AOE@AP hydrogels, 50 mg/mL AHA was dissolved in deionized water to obtain the AHA solution. 16 mg/mL EGCG, 74 mg/mL OSA‐PBA and 10 mg/ml PLLA+APF (PLLA and APF, respectively. m/m = 1/1) were dispersed and dissolved in deionized water to obtain the (EGCG+OSA‐PBA+PLLA+APF) solution. The AOE@AP hydrogel was obtained by mixing the two solutions evenly with same volume in a 2 mL centrifuge tube and standing at 37°C. The hydrogel was named AOE@AP5.In addition, the preparation of AOEF@AP hydrogels is as followed: 50 mg/mL AHA and 1 mg/mL F‐SAP was dissolved in deionized water to obtain the (AHA+F‐SAP) solution. The 16 mg/mL EGCG, 74 mg/mL OSA‐PBA, and 2, 10, 20 mg/ml PLLA+APF (PLLA and APF, respectively. m/m = 1/1) were dissolved or dispersed in deionized water to obtain the (EGCG+OSA‐PBA+PLLA+APF) solution with the 0.2%, 1%, and 2% concentration of PLLA. The AOEF@AP hydrogel was obtained by mixing the two solutions with same volume evenly in a 2 mL centrifuge tube and standing at 37°C. The hydrogels obtained after mixing (AHA+F‐SAP) solution with 0.2%, 1%, and 2% (EGCG+OSA‐PBA+PLLA+APF) solutions were named AOEF@AP1, AOEF@AP5, and AOEF@AP10, respectively.

### 4.8 Characterizations

To investigate the chemical and physical characteristics of hydrogel precursor materials and hydrogels, following analyses were performed: ^1^H NMR spectroscopy, Fourier‐transform infrared (FT‐IR) spectroscopy, gelation time test, rheological tests, scanning electron microscopy (SEM), swelling ratio test, in vitro degradation tests, tissue adhesion strength tests, atomic force microscope (AFM), conductivity test, and peptide release test. Details are provided in the Materials and Methods section of the Supporting Information.

### 4.9 In Vitro F‐SAP Release Study

In order to investigate the release kinetics of F‐SAP from the AOEF@AP5 hydrogel under US stimulation, we conducted an ELISA experiment to measure the content of F‐SAP released over time. Details are provided in the Materials and Methods section of the Supporting Information.

### 4.10 Cell Assays

In order to determine the concentration of components in the hydrogel and to verify the effects of the AOEF@AP5 hydrogel on neurons and macrophages, we first extracted the DRG neurons from mice for culturing. Then, the hydrogel acted on DRG neurons and/or RAW264.7 macrophages, we conducted further clarification through CCK8 assay, live/dead staining, immunofluorescence staining, bulk RNA‐seq, qRT‐PCR assay (Table S1), transwell assay, and cell migration assay. Details are provided in the Materials and Methods section of the Supporting Information.

### 4.11 Animal Experiments

In order to further verify the effect of the hydrogel on diabetic wounds, we established diabetic wound models in mice and rabbits, and verified it through experiments such as fixed‐time‐point photography, H&E staining, Masson staining, Sirius red staining, immunofluorescence staining, bulk RNA‐seq, and immunohistochemistry staining. Details are provided in the Materials and Methods section of the Supporting Information. All animal experiments comply with the National Research Council's Guide for the Care and Use of Laboratory Animals. All animal experiments are approved by the Animal Ethics Committee of the Fourth Military Medical University (20241369).

### 4.12 Statistical Analyses

Before data processing, transformation, normalization, or evaluation of outliers are carried out as needed. The data from three individual experiments are expressed as the means ± standard deviations (SD). The sample size (n) for each statistical analysis is indicated in the legend of each graph. One‐way analysis of variance (ANOVA) with Tukey's post hoc test was performed for pairwise comparisons. To compare the means, two groups were analyzed with independent sample *t* tests. The value of *p* < 0.05 was considered to indicate statistical significance. GraphPad Prism was used for statistical analysis.

## Author Contributions

Conceptualization: KW, SWZ, XZ, and BQS. Investigation: KW, SWZ, XZ, SJL, SY, DJ, and YDC. Methodology: KW, SWZ, XZ, SY, JLD, and TW. Data curation: KW, SWZ, XZ, ZY, BLG, BYS, and JAC. Formal analysis: KW, SWZ, XZ, and JAC. Validation: KW, SWZ, BYS, and JAC. Funding acquisition: XZ, BLG, and BQS. Supervision: XZ, BLG, ZY, and BQS. Project administration: XZ, BLG, and BQS. Resources: XZ, BLG, and BQS. Writing – original draft: KW, SWZ, XZ, BYS, and JAC. Writing – review and editing: KW, SWZ, XZ, BYS, and JAC.

## Conflicts of Interest

The authors declare no conflicts of interest.

## Supporting information




**Supporting File**: advs76721‐sup‐0001‐SuppMat.docx.

**Supporting File**: advs76721‐sup‐0002‐MovieS1‐S2.zip.

## Data Availability

All data needed to evaluate the conclusions in the paper are present in the paper and/or the Supplementary Materials. The RNA‐seq data and other data supporting the findings of this study are available from the corresponding author upon reasonable request for non‐commercial research and reproducibility purposes, subject to no redistribution.
