# Supplementary material for: Ultrasound‐Activated Piezoelectric Neuroimmune Hydrogel Orchestrates Neurogenesis‐Macrophage Crosstalk in Diabetic Wound Healing
Source: Adv Sci (Weinh). 2026 Jul 28:e76721. Online ahead of print. doi: 10.1002/advs.76721 (PMC13410797; doi:10.1002/advs.76721)
Supplement: Supplementary file 1 — Supporting File: advs76721‐sup‐0001‐SuppMat.docx. [file ADVS-9999-e76721-s001.docx]

**Supplementary Materials**

**Ultrasound-Activated Piezoelectric and Adaptive Hydrogel** **Drivers Neurogenesis-Macrophage Reprogramming Crosstalk for Diabetic Wound Healing**

Kai Wang^a, d1^, Shaowen Zhuo^b1^, Binyu Song^a1^, Jinan Chen^d1^, Xin Zhao^b, c*^, Sijia Li^a^, Shuang You^a^, Dong Jiang^d^, Yuedong Chen^d^, Juanli Dang^a^, Tong Wang^a^, Baolin Guo^b*^, Zhou Yu^a*^, Baoqiang Song^a*^

^a^ Department of Plastic Surgery, Xijing Hospital, The Fourth Military Medical University, Xi'an 710032, China

^b^ State Key Laboratory for Mechanical Behavior of Materials, and Frontier Institute of Science and Technology, Xi'an Jiaotong University 710049, Xi’an, China

^c^ State Key Laboratory of Oral & Maxillofacial Reconstruction and Regeneration, The Fourth Military Medical University, Xi'an, 710032, China

^d^ Diabetic Foot Center, The Air Force Hospital of Eastern Theater of PLA, Nanjing, 210000, China

^1^ These authors contributed equally to this work.

^*^ To whom correspondence should be addressed.

E-mail: zhaoxinbio@mail.xjtu.edu.cn; baoling@mail.xjtu.edu.cn; yz20080512@fmmu.edu.cn; songbq@fmmu.edu.cn

**1. Materials and methods**

**1.1 Characterizations**

**^1^H NMR**

The ^1^H nuclear magnetic resonance (^1^H NMR) (JEOL, JNM-ECZ400S/L1) of AHA, SA-PBA and OSA-PBA were recorded at 25 ℃ using D_2_O as solvent.

**FT-IR**

The FT-IR spectra of AHA, OSA-PBA, AHA/OSA-PBA/EGCG and AHA/OSA-PBA/EGCG/APF/F-SAP were recorded on a Nicolet iS50 FT-IR spectrometer in the 4000–600 cm^-1^ range.

**SEM**

After all the hydrogel samples were sprayed with a thin gold layer, the morphology of PLLA nanofibers, freeze-dried AHA/OSA-PBA/EGCG/F-SAP hydrogels and AHA/OSA-PBA/EGCG/APF/F-SAP@PLLA hydrogels were examined by a field emission scanning electron microscope (Carl Zeiss (Shanghai), GeminiSEM 500).

**AFM**

The morphology and surface potential of PLLA nanofibers were characterized by an atomic force microscopy (Bruker Dimension ICON), and their piezoelectric properties were also tested.

**Rheological test**

A TA rheometer (DHR-2) was employed to test rheological properties of these hydrogels. 500 μL of hydrogel precursor solutions were placed between the parallel plates of 20 mm diameter and with a gap of 1000 μm to form hydrogel *in situ* subsequently. At the constant frequency of 10 rad/s and 1% strain, the time sweep tests of four groups of hydrogel samples were performed at 25 ℃. At 25℃ and strains of 1%, 5% and 10%, frequency scanning tests were performed on four groups of hydrogels.

Completely gelled hydrogel with a 20 mm diameter and a thickness of 1000 µm was placed between 20 mm parallel plates with a gap of 1000 µm at 25 ℃. Then, the alternate step strain sweep test was performed at a ﬁxed angular frequency (10 rad/s) at 25 ℃. Amplitude oscillatory strains were switched from small strain (γ=1%) to subsequent large strain (γ=500%) with 30 s for every strain interval.

**Swelling and degradation test**

The weight remaining ratio (%) of the hydrogels was determined by swelling-degradation tests. Four groups of hydrogel bulks with the same volume (300 μL) were immersed in 1 mL PBS (pH=7.4) at constant temperature (37 ℃) in a shaker with 100 rpm, respectively. At the predetermined time point, hydrogel samples were taken out and rinsed with H_2_O to remove excess salinity. Following that, the hydrogels were weighed. All the tests were repeated 3 times.

The weight remaining ratio (%) of the hydrogels was defined by the following equation:

Weight remaining ratio (%) = W_t_/ W_0_$\times$100%

The equilibrium swelling ratio of hydrogels was defined by the following equation:

Equilibrium swelling ratio (%) = (W_t_-W_0_)/ W_0_$\times$100%

where W_t_ and W_0_ are the wet weight of the remaining hydrogels after degradation at different time points, and the wet weight of the initial hydrogels, respectively.

The average degradation rate of hydrogels was defined by the following equation:

Average degradation rate (mg/h) = (W_i_-W_f_)/ T$\times$100%

where W_f_ and W_i_ are the wet weight of the remaining hydrogels after whole degradation process and the weight of the initial hydrogels, respectively. T is the total degradation time.

**Adhesion strength test**

The tissue adhesion strength of the hydrogels was evaluated by using pig skin via a lap shear test. Pigskin was cut into 10 mm × 30 mm rectangular strips and then sprinkled with 100 μL of hydrogel precursor solution, with the area maintained at 10 mm × 10 mm. Another piece of pig skin of the same size was then pressed against one side of the solution. After pressing the pig skin-sample under a 200 g weight in an 37℃ shaker for 2 h, peeling measurements were performed using an Instron material testing system (MTS Criterion 43, MTS Criterion) at a constant speed of 5 mm/min. All experiments were performed at room temperature. The measurements were repeated 6 times for each sample.

**Measurement of electrical conductivity**

According to the previous research of our group, the hydrogel sample were processed into cuboid with a 30 mm in length, and a 10 mm in width and thickness. The conductivity of different hydrogels was measured using a digital multimeter (Agilent) and the data were recorded. The conductivity was calculated according to the following equation:

1/ρ=L/RS

where L was the length of the hydrogel; R was the resistance of the hydrogel; and S was the cross-sectional area of the hydrogel.

All the tests were conducted 6 times for each set of samples.

**1.****2 In Vitro F-SAP release study**

To prepare hydrogels with the same volume (300 μL), the concentrations of F-SAP and PLLA were set at 0.5 mg/mL and 5 mg/mL, respectively. The hydrogels were divided into three groups: AOEF@AP5, AOEF@AP5+US, and AOE@AP5. Then the hydrogels were placed in EP tubes, and 1.5 mL of deionized water was added to each tube. Subsequently, the samples were subjected to ultrasound treatment at a power of 0.5 W/cm² (40 kHz, 10 min) each day. Each day, 1 mL of the solution was collected, and an equal volume of fresh deionized water was added to maintain the total volume. This process was repeated for continuously 7 days. After the collection was completed, the release amounts were quantified using an ELISA kit (KeyGEN BioTECH, China).

**1.3 Extraction and culture of DRG neuron**

The extraction of dorsal root ganglia (DRG) from neonatal C57BL/6 mice involves a series of meticulous steps to ensure the viability of the neurons for subsequent culture. The procedure begins with the euthanasia of the mouse through cervical dislocation, followed by disinfection using pre-cooled 75% ethanol for 3 to 5 minutes. A midline incision is made along the dorsal surface to separate the skin and underlying fascia and muscles. Under a stereomicroscope, the spinal column is accessed by carefully opening the vertebral canal, allowing for the exposure of the intervertebral foramina where the DRG are located. The DRG, characterized as yellowish nodular structures, are extracted using fine forceps, with the nerve fibers at both ends being severed. The surrounding epineurium is removed, and the ganglia are placed in a culture dish pre-coated with poly-L-lysine. Subsequently, a complete neuronal culture medium is added to facilitate the growth of the neurons. This extraction process is critical for studying sensory neuron physiology and can be adapted for various experimental applications in neuroscience research. All animal experiments comply with the National Research Council's Guide for the Care and Use of Laboratory Animals. All animal experiments are approved by the Animal Ethics Committee of the Fourth Military Medical University (20241369).

**1.4 Cell immunofluorescence analysis**

Macrophages (1×10^4^ cells/well) and DRG explants (10/well) in each group, were fixed with 4% paraformaldehyde for 30 min and then incubated with 0.3% Triton and 5% BSA for 10 min and 1 h, respectively. After the primary antibody (anti-CD86 and anti-206, 1:400, Abcam, USA; anti-β3-tubulin and F-actin, 1:500, Abcam, USA) was incubated at 37 ℃ overnight, the secondary antibody was incubated for 2 h. Nuclei was stained with DAPI for CLSM analysis (Nikon, Japan).

**1.5 qRT-PCR assay**

The total RNA of the cells (1×10^6^ cells/well) co-cultured with different hydrogels was extracted by Trizol method and reversely-transcribed into cDNA using reverse transcription kit (Yeasen, China). According to the operation manual, qRT-PCR analysis was performed using SYBR Green Master Mix (Yeasen, China) and the relative expression was calculated. The primers used in the above experiments are listed in Table S1 of the supplementary information.

**1.6 Biocompatibility evaluation**

The activity of hydrogel was evaluated using the CCK-8 assay kit (KeyGEN BioTECH, China). The hydrogel was sterilized by UV irradiation for 30min, and then the extract was obtained after being added to the DMEM (Hyclone, USA) medium and cultured at 37 ℃ for 24 hours. Macrophages (1×10^3^ cells/well) were inoculated into 96-well plates at a density of 1×10^3^/ well, incubated overnight and replaced with extracts from the previous medium. According to the operating manual, after adding 10 μL of CCK-8 working liquid, the absorbance at 450 nm per well was measured with an enzyme-labeled meter. In addition, the cytotoxicity of the hydrogel was evaluated by a live cell/dead cell staining kit (calcein-AM/Propyl iodide, Beyotime, China), in which live cells were colored green by calcein-AM and dead cells were colored red by propyl iodide for CLSM analysis (Nikon, Japan).

**1.7 Assessment of macrophage polarization *in vitro***

In brief, RAW 264.7 macrophages (1×10^6^ cells/well) were seeded and cultured in the lower chamber of transwell for 24 h, and then the 100 μL hydrogel was placed in the upper chamber and 200 μL of medium were added. After 48 h, the relative expression of pro-inflammatory (IL-1β, iNOS, and TNF-𝛼) and anti-inflammatory (IL-4, IL-10, and Arg-1) genes were assessed using qRT-PCR. The relative expression of pro-inflammatory (iNOS) and anti-inflammatory (CD206) proteins were assessed using immunofluorescence staining.

**1.8 Assessment of DRG neuron regeneration *in vitro***

In brief, DRG explants (30/well) were seeded and cultured in the lower chamber of transwell for 24 h, and then the 100 μL hydrogel was placed in the upper chamber and 200 μL of medium were added. After 48 h, the relative expression of (GAP43, NF, Syn-1, and TUJ1) genes were assessed using qRT-PCR. The relative expression β3-tubulin of were assessed using immunofluorescence staining.

**1.9 Cell migration assay**

Transwell assays were performed to evaluate cell migration. Macrophages/PC12 cells (5×10^4^ cells/well) were added to the upper chamber (8 μm) in 500 μL of DMEM/RPMI 1640 medium supplemented with serum-low medium. Approximately 600 μL of culture supernatant from the various samples was added to the lower chamber. After cultivation in an incubator at 37°C for 48 h, the cells were stained with crystal violet.

**1.10 Migration assay**

PC12 cells were seeded at 1 × 10^6^ cells per well in six-well plates. A straight scratch on the monolayer cell was created using a 20 μL sterile pipette tip after the PC12 cells reached 80 to 90% confluency, and the scraped cells were gently washed with PBS. Then, hydrogel was added and incubated with the cells to observe the effect of hydrogel on wound healing. The healing ratio of the scratch area was quantified by ImageJ software.

**1.11 Diabetic mouse wound healing evaluation *in vivo***

Briefly, in order to construct an animal model of type Ⅱ diabetes, 8-week female mice were fed by high-fat and high-sucrose diet for 1 month, followed by intraperitoneal injection of streptozotocin (STZ, 1%) at three consecutive days under isoflurane anesthesia. Blood glucose levels were measured 72 h and at 3-day intervals after STZ injection. A blood glucose level higher than 16.7 mM indicated the successful construction of type Ⅱ diabetes model. The hair on the mice back was shaved the day before the diabetic wound model was constructed. After the mouse were anesthetized by isoflurane, a biopsy punch was used to create two full-thickness skin wounds with a diameter of about 1 cm in the back skin. It is divided into 6 groups: Control, US, AOEF, AOEF+US, AOEF@AP5, and AOEF@AP5+US. About 100 μL of the hydrogel was applied to the wound to assess its therapeutic efficacy on wound healing. Subsequently, the samples of US, AOEF+US, and AOEF@AP5+US were subjected to ultrasound treatment at a power of 0.5 W/cm² (40 kHz, 10 min) each two days. In order to avoid the influence of ultrasonic heat generation on the experimental results, the ultrasonic probe does not come into contact with the sample and the skin tissue, but it does not affect the ultrasonic effect. After surgery, isometric photographs of wound healing were taken at days 0, 3, 7, and 14. All animal experiments comply with the National Research Council's Guide for the Care and Use of Laboratory Animals. All animal experiments are approved by the Animal Ethics Committee of the Fourth Military Medical University (20241369).

**1.11 Diabetic rabbits wound healing evaluation *in vivo***

Briefly, in order to construct an animal model of diabetes, 8-week female rabbit were fed by high-fat and high-sucrose diet for 10 days, followed by venae auricularis injection of streptozotocin (STZ, 20 mg/mL, 40 mg/kg) at twice a day for 10 days under anesthesia^1^. Blood glucose levels were measured 72 h and at 3-day intervals after STZ injection. A blood glucose level higher than 11.0 mM indicated the successful construction of diabetes model. The hair on the ear was shaved the day before the diabetic wound model was constructed. After the rabbits were anesthetized, a biopsy punch was used to create six full-thickness skin wounds with a diameter of about 6 mm in the back skin. It is divided into 6 groups: Control, US, AOEF, AOEF+US, AOEF@AP5, and AOEF@AP5+US. About 100 μL of the hydrogel was applied to the wound to assess its therapeutic efficacy on wound healing. Subsequently, the samples of US, AOEF+US, and AOEF@AP5+US were subjected to ultrasound treatment at a power of 0.5 W/cm² (40 kHz, 10 min) each two days. After surgery, isometric photographs of wound healing were taken at days 0, 7, 14, 21, and 28. All animal experiments comply with the National Research Council's Guide for the Care and Use of Laboratory Animals. All animal experiments are approved by the Animal Ethics Committee of the Fourth Military Medical University (20241369).

**1.12 Histology and immunostaining evaluation**

On day 7 and 14 or 28 after surgery, the wound and surrounding tissues were removed directly, and the mice and rabbit were then executed. The tissue samples were fixed with 4% formalin and embedded in paraffin wax, then cut into sections 4 μm thick. For histological examination, sections were analyzed using hematoxylin and eosin (H&E) and Masson’s trichrome staining, where H&E, Masson, and Sirius red staining were viewed under a light microscope, and the results were finally analyzed using the ImageJ software.

For immunofluorescence staining, F4/80 (Abcam, USA, 1:400), iNOS (Abcam, USA, 1:200), CD206 (Proteintech, USA, 1:200), NF (Proteintech, USA, 1:200), and β3-tubulin (Abcam, USA, 1:400) were used to detect the degree of macrophage polarization. CD31 (Proteintech, USA, 1:400) were used to detect vascular regeneration. After staining, the results were observed under a fluorescence microscope and photographed.

**1.13 RNA extraction and library construction**

Full-thickness puncture biopsies were performed on the skin of AOEF@AP5+US wounds and control wounds from 3 wounds of 3 mice on day 14 and macrophages/DRG neurons were treated with AOEF@AP5+US hydrogel and control on 48 h for quantitative RNA sequencing. RNA extraction was carried out using Trizol reagent (Invitrogen, Carlsbad, CA, USA) according to the manufacturer's protocol to isolate and purify total RNA from the wound tissues. The quantity and purity of the extracted RNA samples were assessed using a NanoDrop ND-1000 (NanoDrop, Wilmington, DE, USA). The integrity of the RNA was evaluated using a Bioanalyzer 2100 (Agilent, CA, USA) based on an RNA integrity number (RIN) greater than 7.0. Additionally, electrophoresis with a denaturing agarose gel was performed to confirm RNA integrity.

Poly(A) RNA enrichment was carried out using Dynabeads Oligo (dT) 25-61005 (Thermo Fisher, CA, USA) through two rounds of purification from 1 μg of total RNA. Subsequently, the poly(A) RNA was fragmented using the Magnesium RNA Fragmentation Module (NEB, cat. e6150, USA) at 94°C for 5-7 minutes. The resulting fragmented RNA was reverse-transcribed into cDNA with SuperScript™ II Reverse Transcriptase (Invitrogen, cat. 1896649, USA). To generate U-labeled second-stranded DNAs, *E. coli* DNA polymerase I (NEB, cat. m0209, USA), RNase H (NEB, cat. m0297, USA), and dUTP Solution (Thermo Fisher, cat. R0133, USA) were used. An A-base was then added to the blunt ends of each strand in preparation for ligation to indexed adapters. The adapters contained T-base overhangs for ligating to the A-tailed fragmented DNA. Single- or dual-index adapters were ligated to the fragments, and size selection was performed using AMPureXP beads. After treatment with the heat-labile UDG enzyme (NEB, cat. m0280, USA) to remove the U-labeled second-strand DNA, the ligated products were subjected to PCR amplification under the following conditions: initial denaturation at 95°C for 3 minutes; 8 cycles of denaturation at 98°C for 15 seconds, annealing at 60°C for 15 seconds, and extension at 72°C for 30 seconds; and a final extension step at 72°C for 5 minutes. The resulting cDNA libraries had an average insert size of 300 ± 50 bp. Finally, paired-end sequencing (PE150) was performed on an Illumina NovaSeq™ 6000 platform (LC-Bio Technology Co., Ltd., Hangzhou, China) according to the vendor's recommended protocol.

**1.14 Bulk RNA sequencing data analysis**

The raw reads obtained from the bulk RNA sequencing were initially processed using fastp software (https://github.com/OpenGene/fastp) with default parameters to remove reads containing adaptor contamination, low-quality bases, and undetermined bases. The quality of the processed sequences was further assessed using fastp. To align the clean reads to the *Homo sapiens* GRCh38 reference genome, we employed HISAT2 (https://ccb.jhu.edu/software/hisat2). The mapped reads from each sample were subsequently assembled using StringTie (https://ccb.jhu.edu/software/stringtie) with default settings. Subsequently, all of the transcriptomes generated from individual samples were merged using gffcompare (https://github.com/gpertea/gffcompare/) to reconstruct a comprehensive transcriptome. Following the construction of the final transcriptome, the expression levels of all the transcripts were estimated using StringTie. Specifically, StringTie calculates the Fragments Per Kilobase of transcript per Million mapped reads (FPKM) to quantify mRNA expression levels (FPKM = [total_exon_fragments/mapped_reads(millions)×exon_length(kB)]. Differentially expressed mRNAs were identified using the R package DESeq2, with a significance threshold set at a p value < 0.05. Finally, to gain insight into the biological functions of the differentially expressed genes, Gene Ontology (GO), Kyoto Encyclopedia of Genes and Genomes (KEGG) enrichment analyses, and Gene set enrichment analysis (GSEA) were conducted using the clusterProfiler package.

**2. Results**

**2.1 Screening of EGCG, F-SAP, and PLLA content in hydrogels**

The composition and functionality of hydrogels are intricately linked, making the optimization of component concentrations a crucial step in their development [30]. Our study focused on systematically fine-tuning the concentrations of key components to achieve desired biological effects. We initially explored the optimal concentration of EGCG by formulating hydrogels with varying concentrations of EGCG (4, 5, 8, and 16 mg/mL) in combination with AHA and OSA-PBA. The hydrogels were evaluated for cytotoxicity using live/dead staining and the CCK-8 assay. Results indicated that all tested concentrations exhibited good biocompatibility, with no significant cytotoxicity observed (**Fig. S20A**). Notably, the 8 mg/mL EGCG group demonstrated the highest cell viability, suggesting enhanced cell proliferation (**Fig. S20B**). Furthermore, qRT-PCR analysis revealed that the 8 mg/mL group exhibited superior anti-inflammatory effects, effectively promoting the polarization of M2 macrophages (**Fig. S20C**). Thus, 8 mg/mL was selected as the optimal concentration for EGCG. Next, we optimized the concentration of F-SAP by incorporating different concentrations (0.25, 0.5, 1, and 2 mg/mL) into hydrogels containing AHA, EGCG, and OSA-PBA. The hydrogels were applied to DRG neurons, and axonal regeneration was assessed via immunofluorescence staining (**Fig. S21**). The 0.5 mg/mL F-SAP group showed the most significant axonal regeneration, leading to its selection as the optimal concentration. We further optimized the concentration of PLLA by preparing hydrogels with various PLLA concentrations (1, 5, and 10 mg/mL, with and without ultrasound stimulation) in combination with AHA, EGCG, F-SAP, and OSA-PBA. Subsequently, the samples were subjected to ultrasound treatment at a power of 0.5 W/cm² (40 kHz, 10 min) each day^2^. Previous literature has shown that the magnitude of the piezoelectric effect is closely related to the concentration of PLLA. A too small piezoelectric effect is insufficient to achieve the effect of regulating neurogenesis-macrophage reprogramming, while a too large piezoelectric effect may cause additional damage to cells and tissues^3^. Therefore, further strict screening of the concentration of PLLA is of crucial importance. The hydrogels were tested on DRG neurons and macrophages. Immunofluorescence staining showed that the US+5 mg/mL PLLA group had the best axonal regeneration (**Fig. S22A and S22B**), while qRT-PCR confirmed its efficacy in promoting M2 macrophage polarization (**Fig. S22C**). Therefore, 5 mg/mL was chosen as the optimal PLLA concentration.

After determining the optimal component concentrations, we investigated the impact of ultrasound stimulation on F-SAP release from the hydrogels. In vitro release experiments revealed that ultrasound significantly increased F-SAP release, particularly on the second day, compared to non-ultrasound groups (**Fig. S23**). This accelerated release suggests that ultrasound stimulation can enhance the delivery of bioactive substances, potentially accelerating wound healing. In summary, our study meticulously optimized the concentrations of key hydrogel components to achieve desired biological effects, including cell proliferation, anti-inflammatory activity, and axonal regeneration. The findings highlight the potential of these hydrogels as effective delivery systems for chronic wound healing, with ultrasound stimulation further enhancing their therapeutic efficacy.

**
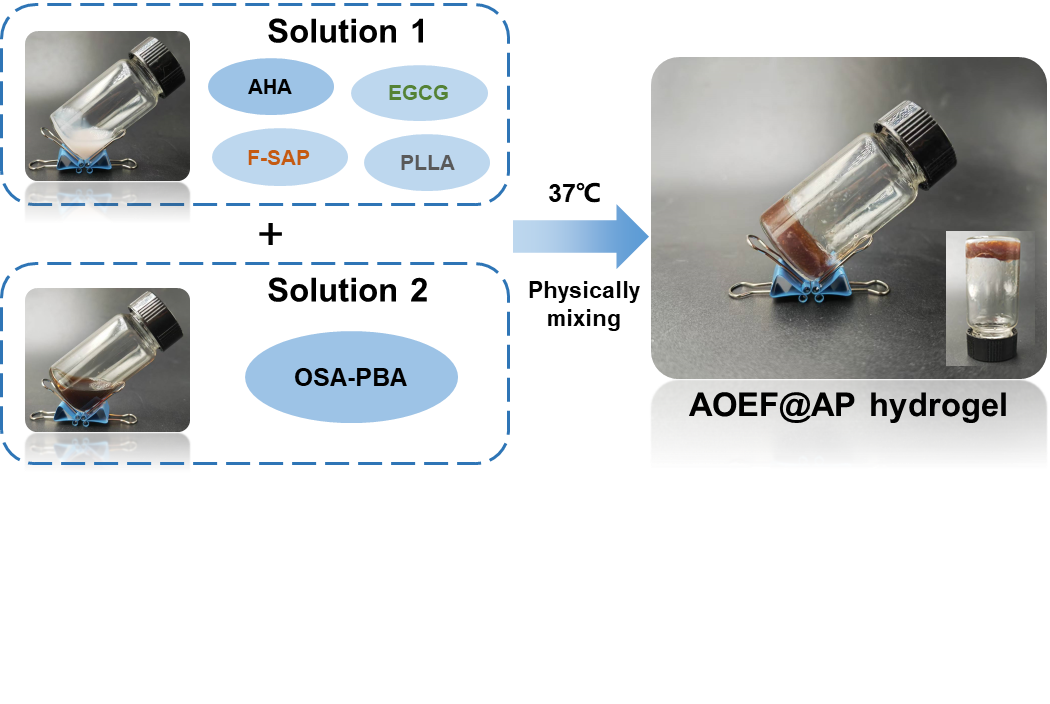
**

**Fig. S1.** Schematic diagram of hydrogel preparation.

**
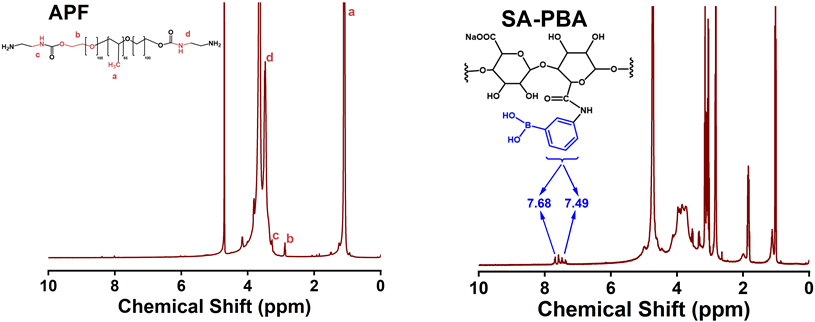
**

**Fig. S2.** ^1^H NMR spectra of APF and SA-PBA.

**
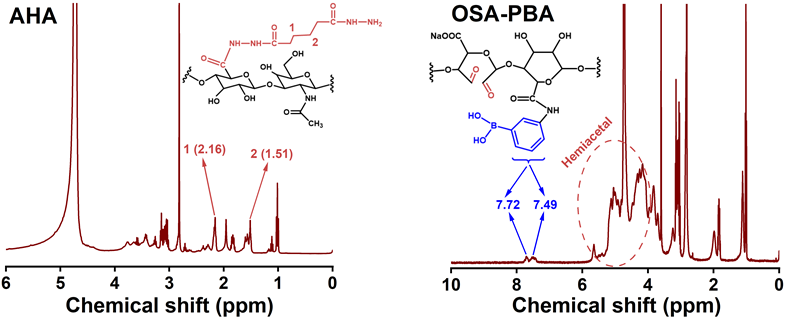
**

**Fig. S3.** ^1^H NMR spectra of AHA and OSA-PBA.

**
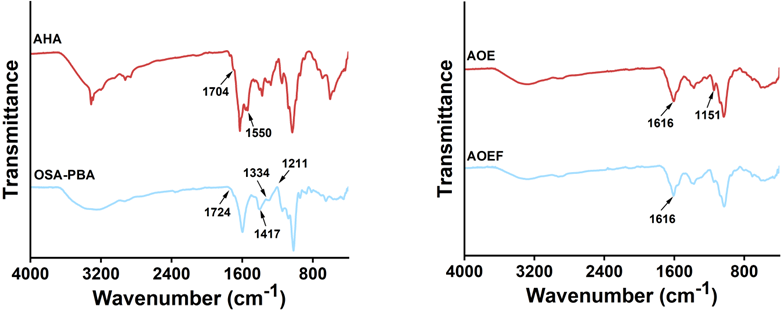
**

**Fig. S4.** FT-IR spectra of AHA, OSA-PBA and hydrogels.

**
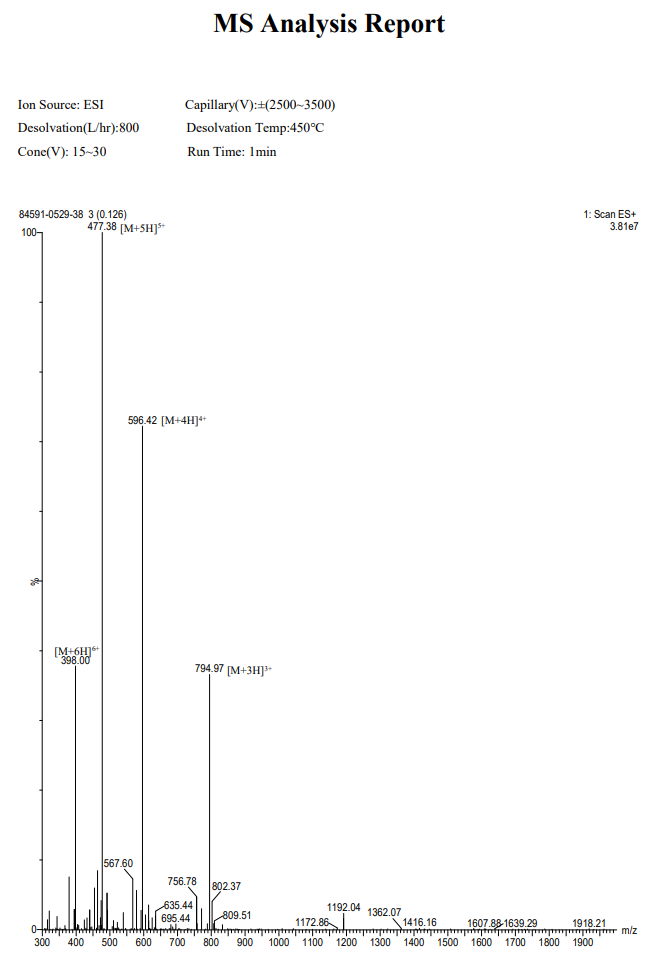
**

**Fig. S5.** Mass spectrum of F-SAP.

**
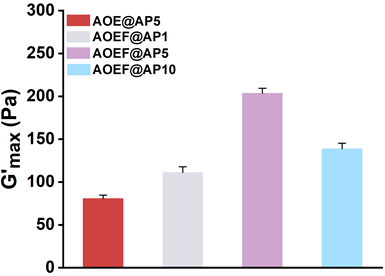
**

**Fig. S6.** Maximum storage moduli of hydrogels (G’max, n=3).


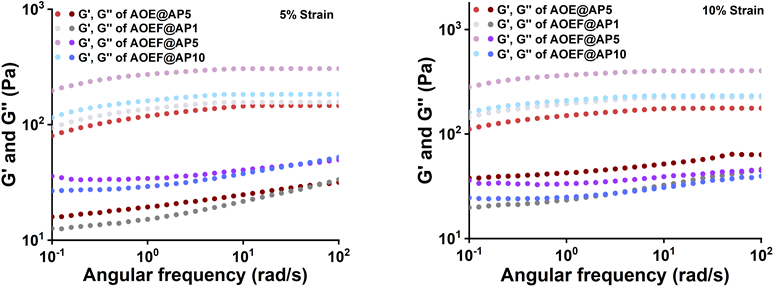


**Fig. S7.** Frequency-dependent rheological behavior of the hydrogels with constant strains of 5% and 10%, and a frequency range of 10^-1^–10^2^ rad/s at 37°C (n = 1).

**
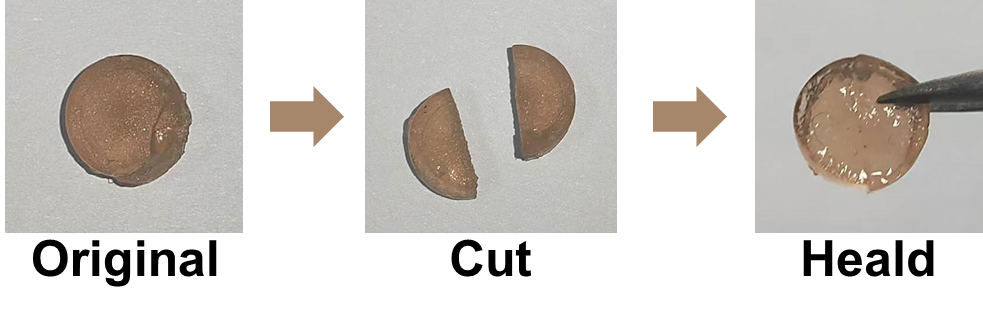
**

**Fig. S8.** Self-healing property of the AOEF@AP5 hydrogel.

**
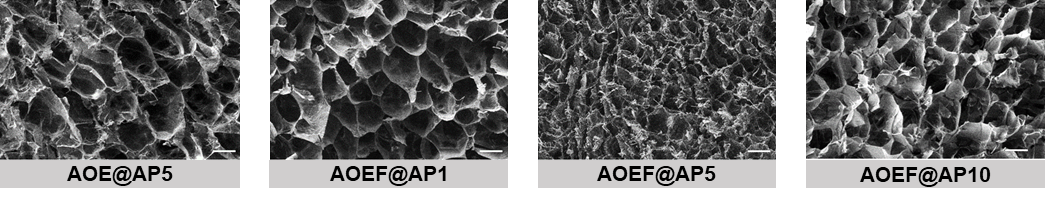
**

**Fig. S9.** Microscopic morphology of hydrogels taken by scanning electron microscope, magnification: 200X, scale bar: 50 μm.

**
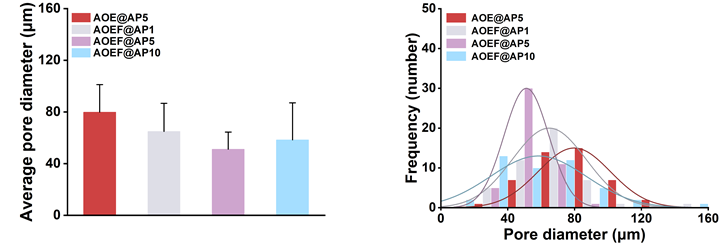
**

**Fig. S10.** Average pore diameter (n=15) and distribution of pore diameters (n=1) of hydrogel.

**
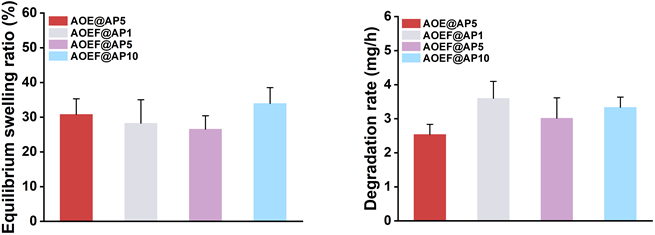
**

**Fig. S11.** Equilibrium swelling ratio (n=3) and average degradation rate (n=3) of the hydrogels.

**A B**

**
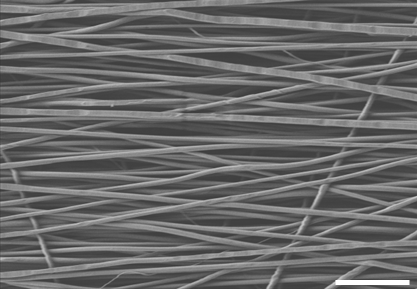

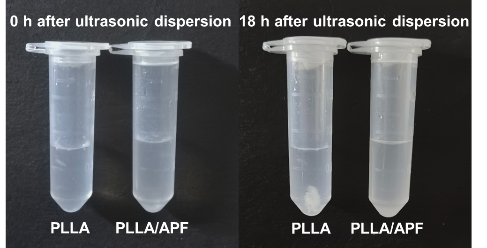
**

**Fig. S12.** (A) SEM image of film PLLA before sectioning, Scale bar: 30 µm. (B) Photograph of PLLA with APF or without APF after ultrasonic dispersion *in vitro*.

**
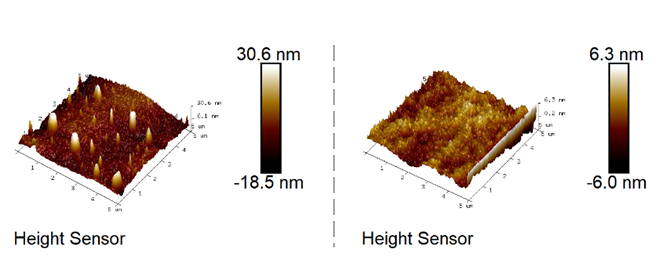
**

**Fig. S13.** 3D surface morphology of unannealed PLLA (left) and annealed PLLA (right), scale bar = 1.0 μm.

**
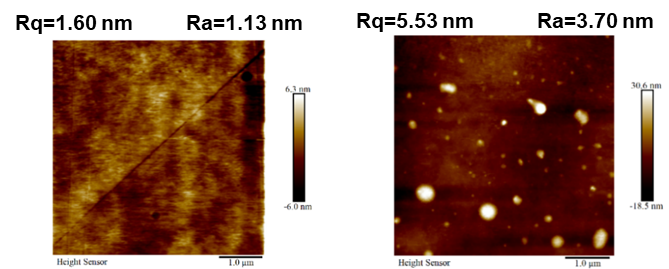
**

**Fig. S14.** Surface roughness distribution of unannealed PLLA (left) and annealed PLLA (right), scale bar = 1.0 μm.

**
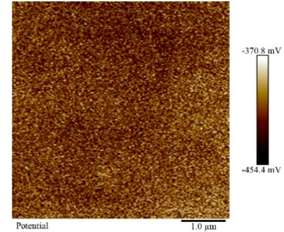
**

**Fig. S15.** Surface potential distribution of annealed PLLA, scale bar = 1.0 μm.

**
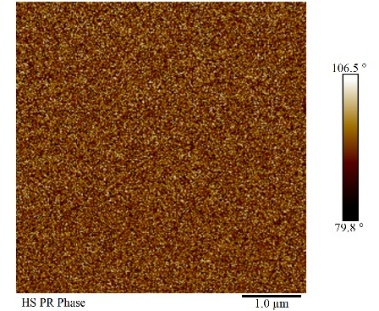
**

**Fig. S16.** Surface phase angle distribution of annealed PLLA, scale bar: 1.0 μm.


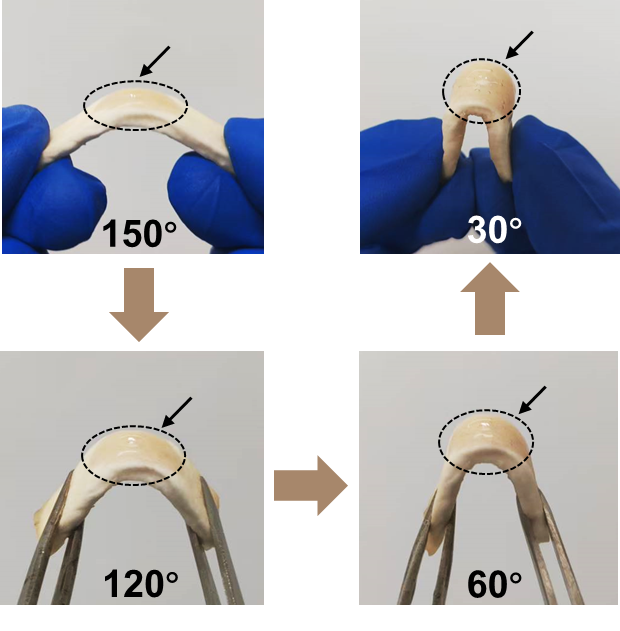


**Fig. S17.** Photograph of adhesion stability of the AOEF@AP5 hydrogel after being bent at different angles.


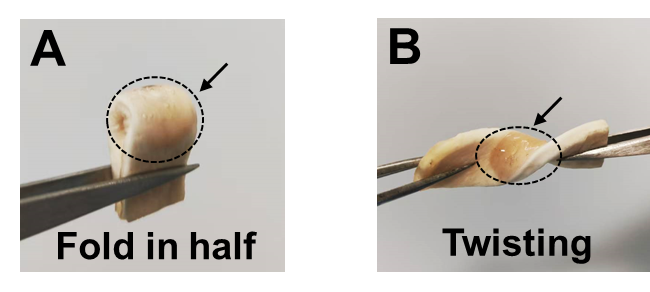


**Fig. S18.** Photograph of adhesion stability of the AOEF@AP5 hydrogel after being folded in half and twisted.


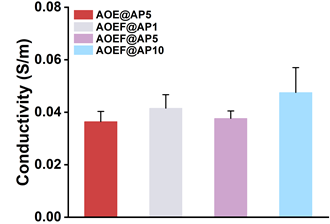


**Fig. S19.** Electrical conductivity of hydrogels.


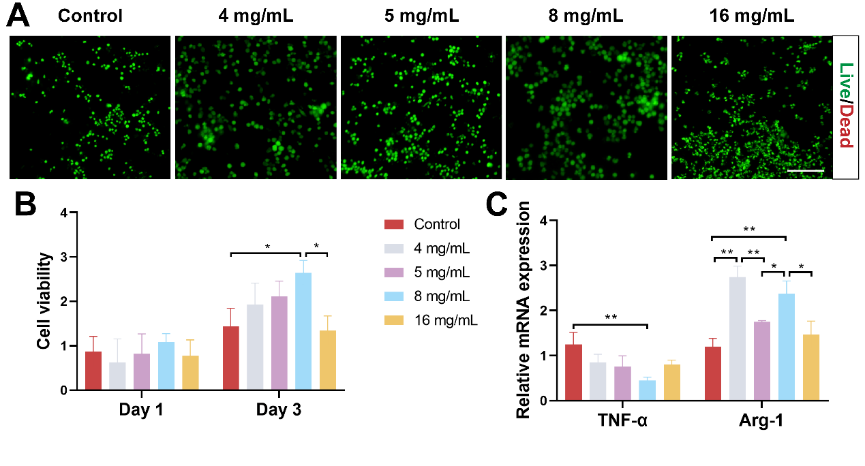


**Fig. S20.** Screening of EGCG with different concentrations. (A) Representative photographs of Live/dead staining on macrophage in different groups. Scale bar: 100 μm. (B) CCK8 assay on macrophage in different groups. (C) qRT-PCR assay detecting the expression levels of pro-inflammatory cytokines TNF-α and anti-inflammatory cytokines Arg-1. (* *p* < 0.05, ** *p* < 0.01)


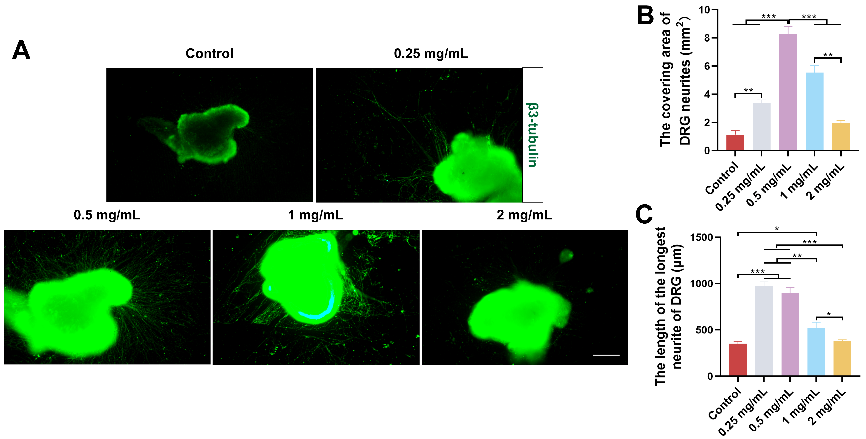


**Fig. S21.** Screening of F-SAP with different concentrations. (A) Representative immunofluorescence photographs of β3-tubulin staining on DRG neurons in different groups. Scale bar: 500 μm. (B) Quantitative analysis of the covering area of DRG neurites in different groups. (C) Quantitative analysis of the length of the longest neurite of DRG in different groups. (* *p* < 0.05, ** *p* < 0.01, *** *p* < 0.001)


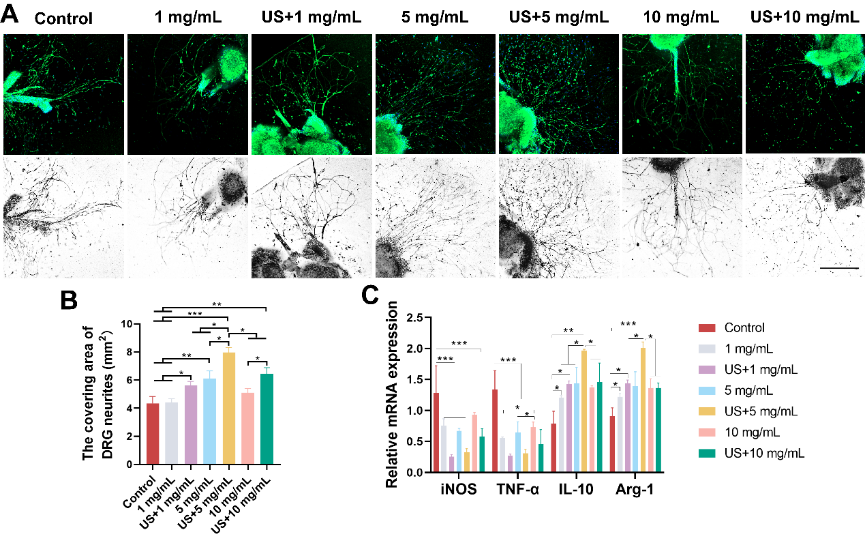


**Fig. S22.** Screening of PLLA with different concentrations. (A) Representative immunofluorescence photographs of β3-tubulin staining on DRG neurons in different groups. Scale bar: 500 μm. (B) Quantitative analysis of the covering area of DRG neurites in different groups. (C) qRT-PCR assay detecting the expression levels of pro-inflammatory cytokines iNOS and TNF-α and anti-inflammatory cytokines IL-10 and Arg-1. (* *p* < 0.05, ** *p* < 0.01, *** *p* < 0.001)


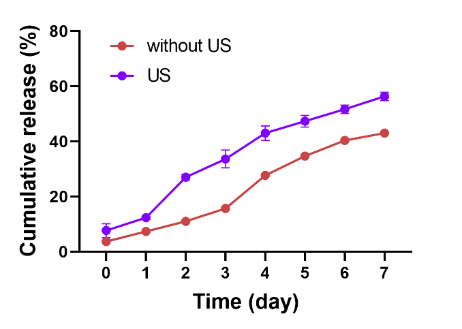


**Fig. S23.** F-SAP release of AOEF@AP5 hydrogels with US or without US *in vitro*.


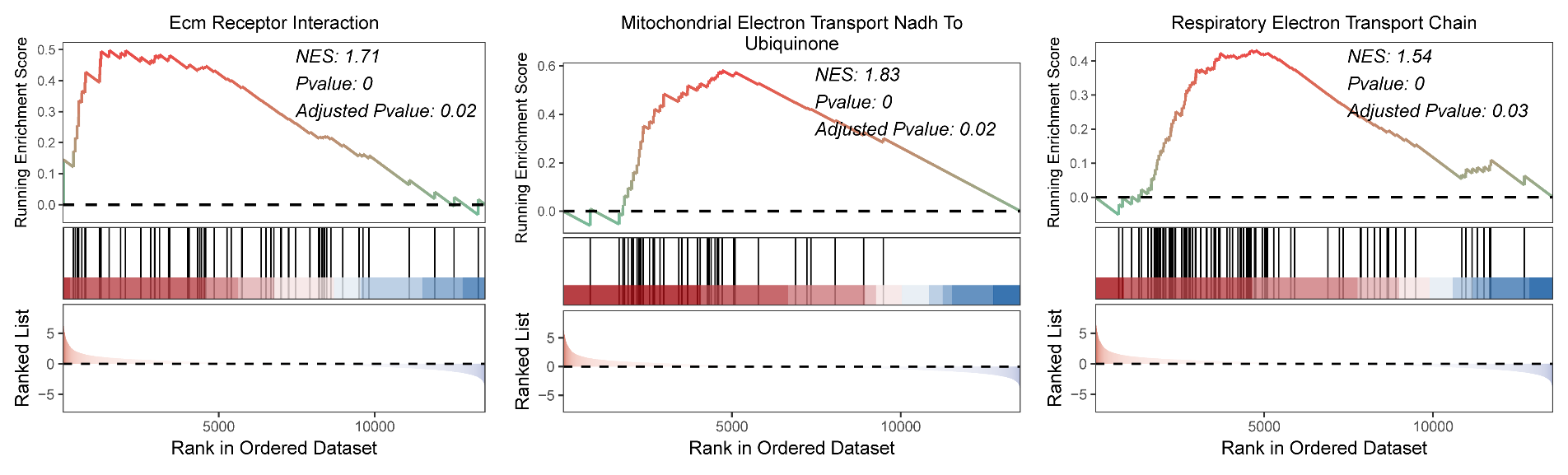


**Fig. S24.** GSEA of AOEF@AP5+US hydrogel group on macrophages.


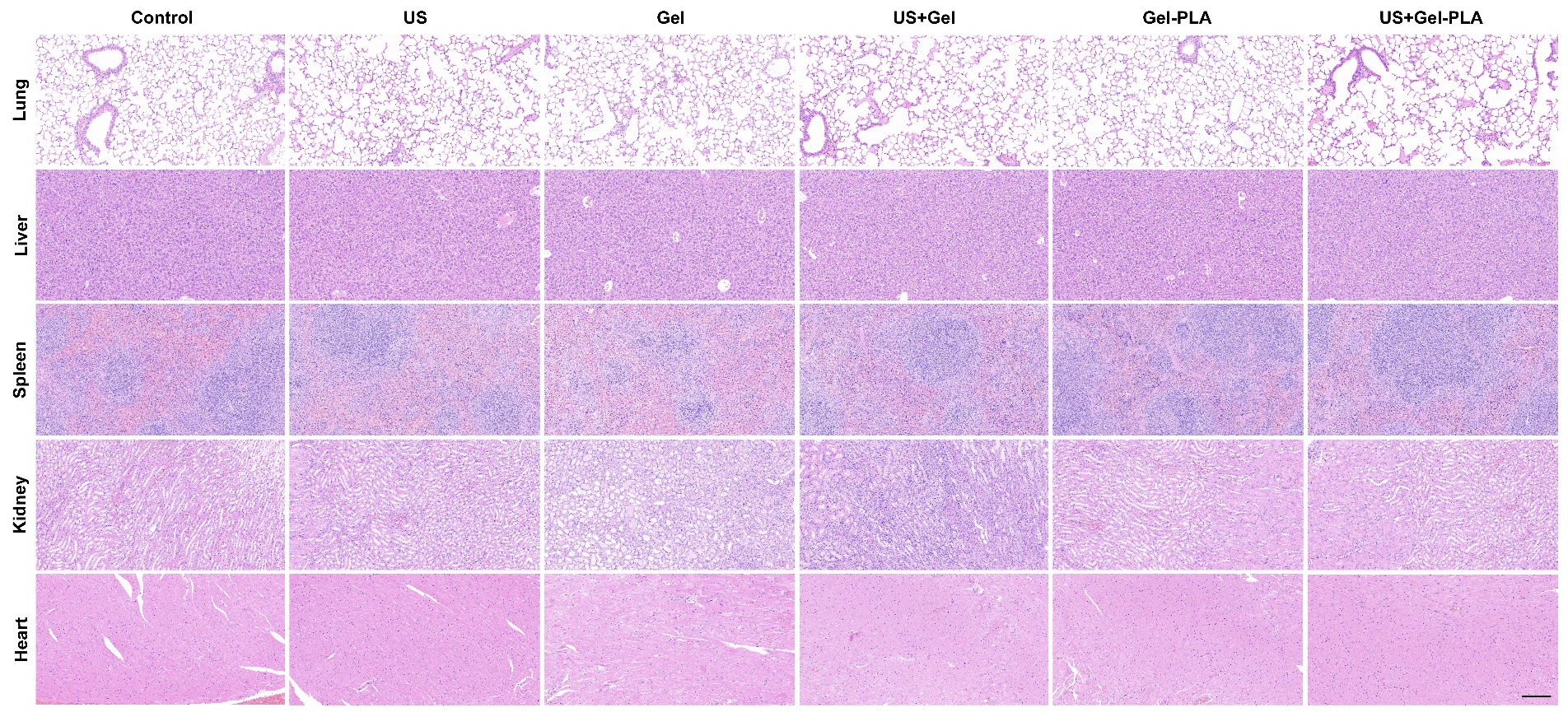


**Fig. S25.** H&E staining of important organs of mice 14 days after wound in different groups.


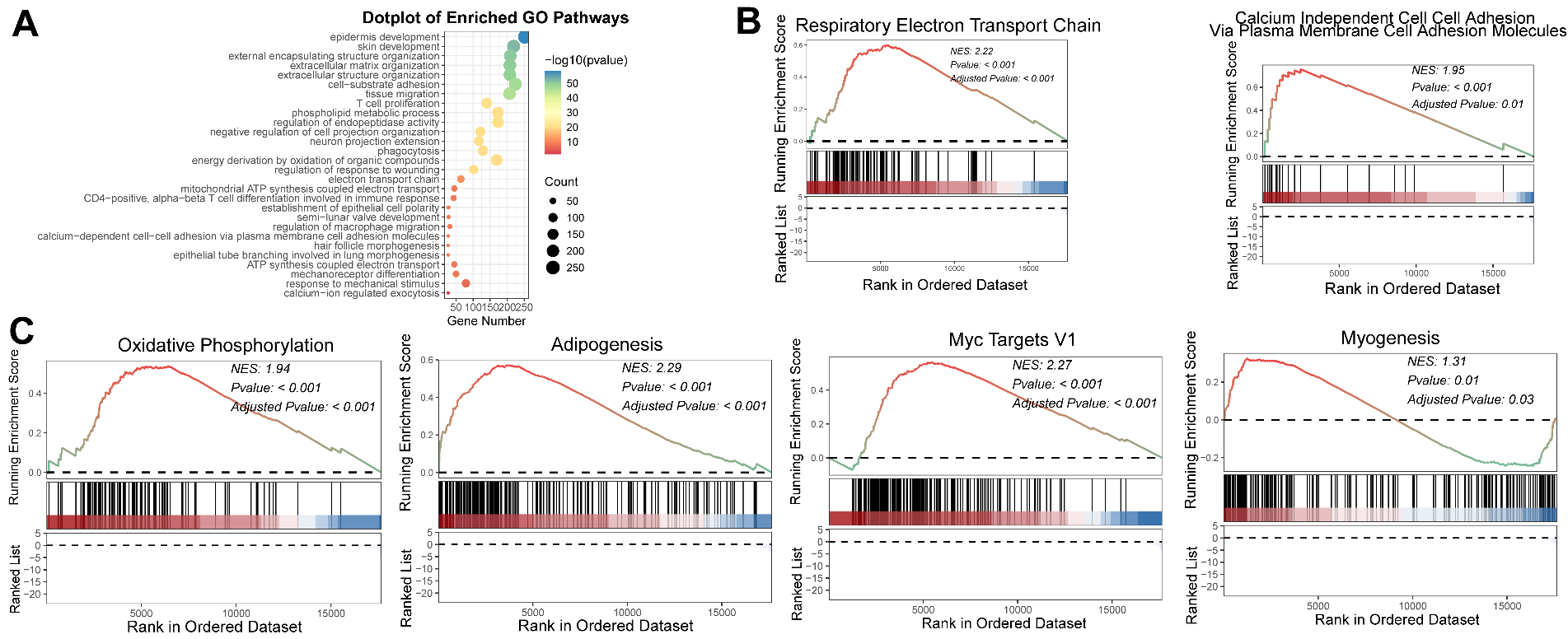


**Fig. S26.** GO enrichment analysis (A) and GSEA (B and C) of AOEF@AP5+US hydrogel group on 14 days after wound.

**Table**

**Table S1 Primers used in the qRT-PCR analysis**

| **Gene** | **Forward Primer (5’ to 3’)** | **Reverse Primer (5’ to 3’)** |
| --- | --- | --- |
| GAPDH | AGACAGCCGCATCTTCTTGT | CTTGCCGTGGGTAGAGTCAT |
| TUJ1 | CCCGTTTTAGCCACCTTTGTATT | CCCTCCAAATATAAACACAACCC |
| Syn-1 | CTTCCTGGTTGGGGACTACTCCTC | GCGAACACGGCTGTAGCCAGAAAG |
| NF | GTTCCGAGTGAGGTTGGACC | CCGCCGGTACTCAGTTATCTC |
| GAP43 | GCACATCGGCTTGTTTAGGCT | GGAGGGAGATGGCTCTGCTACT |
| iNOS | CAAGCTGAACTTGAGCGAGGA | TTTACTCAGTGCCAGAAGCTGGA |
| TNF-α | CCCTCACACTCAGATCATCTTCT | GCTACGACGTGGGCTACAG |
| IL-1β | TCCAGGATGAGGACATGAGCAC | GAACGTCACACACCAGCAGGTTA |
| Arg-1 | AGCTCTGGGAATCTGCATGG | ATGTACACGATGTCTTTGGCAGATA |
| IL-4 | CCATATCCACGGATGCGACA | AAGCCCGAAAGAGTCTCTGC |
| IL-10 | GCTCTTACTGACTGGCATGAG | CGCAGCTCTAGGAGCATGTG |

**Supplementary notes**

**Supplementary note 1: Characterization of AOEF@AP Hydrogels**

In the ^1^H NMR spectrum of AHA, characteristic peaks at 2.16 ppm and 1.51 ppm corresponded to protons between the two carbonyl groups, indicating successful ADH conjugation (**Fig. 1B**). For SA-PBA, peaks at 7.68 and 7.49 ppm arose from aromatic protons of the PBA moiety (**Fig. S1**). After oxidation, OSA-PBA retained aromatic signals at 7.72 and 7.68 ppm, while new peaks between 4-6 ppm appeared, attributable to hemiacetal protons formed by oxidized aldehydes and neighboring hydroxyls (**Fig. 1C**). In the ^1^H NMR spectrum of APF, the signal assigned to terminal methyl groups (peak a) and the -OCH₂ protons adjacent to the carbonate (peak b), together with amide-related signals (peaks c and d), confirmed successful amination of PF127 (**Fig. S2**). FT-IR spectra further supported the successful synthesis of the main precursors. AHA showed characteristic amide C=O stretching at 1710 and 1550 cm⁻¹, while OSA-PBA displayed a weak band at 1724 cm⁻¹ corresponding to aldehyde groups, together with peaks at 1417 and 1211 cm⁻¹ (aromatic ring vibrations) and at 1334 cm⁻¹ (boronic group vibration) (**Fig. 1D**). The mass spectrum of F-SAP exhibited dominant peaks at m/z 398.00, 477.38, 596.42 and 794.97, corresponding to the 3+, 4+, 5+ and 6+ charge states of the peptide, confirming its expected molecular weight (**Fig. S3**). Hydrogel formation was achieved at 37 °C through multi-dynamic crosslinking among AHA, OSA-PBA, EGCG and F-SAP (**Scheme**). Phenylboronic acid groups on OSA-PBA formed boronate ester bonds with catechols on EGCG, while aldehydes on OSA-PBA simultaneously formed acylhydrazone bonds with hydrazides on AHA. In parallel, primary amines on F-SAP reacted with remaining aldehydes to generate imine (Schiff base) linkages. In the FT-IR spectrum of the formed hydrogels, absorption bands around 1616 cm⁻¹ and 1151 cm⁻¹ were consistent with the presence of boronate esters, acylhydrazones and imines (**Fig. 1E**), confirming successful construction of the multi-dynamic covalent network that underpins injectability, self-healing and tissue adhesion.

**Supplementary note 2: Screening and determination of concentrations of key components in hydrogels**

Hydrogels containing AHA and OSA-PBA were prepared with increasing EGCG concentrations (4, 5, 8 and 16 mg mL⁻¹). Based on live/dead staining, CCK-8 assays and qRT-PCR of inflammatory markers, 8 mg mL⁻¹ EGCG was selected as the optimal dose that balanced cytocompatibility with anti-inflammatory potential (**Fig. S20**). We next tuned F-SAP loading (0.25, 0.5, 1 and 2 mg mL⁻¹) in AHA/OSA-PBA/EGCG hydrogels and evaluated dorsal root ganglion (DRG) neurite outgrowth. Immunofluorescence analysis showed that 0.5 mg mL⁻¹ F-SAP produced the most pronounced axonal extension, and this concentration was therefore chosen for subsequent studies (**Fig. S21**). The PLLA content was then optimized with and without US stimulation. Hydrogels containing 1, 5 or 10 mg mL⁻¹ PLLA (with fixed AHA, OSA-PBA, EGCG and F-SAP) were tested on DRG neurons and RAW264.7 macrophages. Immunofluorescence staining and qRT-PCR demonstrated that the US-treated 5 mg mL⁻¹ PLLA group yielded the best combination of neurite outgrowth and pro-regenerative macrophage phenotype (**Fig. S22**). On this basis, 5 mg mL⁻¹ was defined as the working concentration of PLLA. Finally, we examined the effect of US on F-SAP release from the optimized hydrogel. In vitro release experiments showed that US significantly enhanced cumulative F-SAP release from AOEF@AP5, with a marked increase on day 2 compared with non-irradiated hydrogels (**Fig. S23**), confirming that US provides an effective trigger to modulate neuropeptide availability. For clarity, the following nomenclature is used throughout the study: AOE@AP5 (AHA/OSA-PBA/EGCG/APF@PLLA5 hydrogel, no F-SAP, no US); AOE@AP5+US (AOE@AP5 subjected to ultrasound stimulation); AOEF@AP5 (AHA/OSA-PBA/EGCG/APF/F-SAP@PLLA5 hydrogel, with F-SAP, no US); AOEF@AP5+US (AOEF@AP5 under ultrasound activation).

**Supplementary notes 3: Microstructure, Swelling and Degradation** **Behavior of Hydrogels**

SEM images of freeze-dried samples showed that all four formulations exhibited loose, interconnected porous networks (**Fig. 1G**, **Fig. S9**). Quantitative analysis revealed average pore diameters below 80 μm in all groups from 79.83 μm (AOE@AP5) to 64.87 μm (AOEF@AP1), 51.18 μm (AOEF@AP5) and 58.38 μm (AOEF@AP10). Pore-size distribution analysis (**Fig. S10**) showed that all hydrogels had pores mainly in the 40-120 μm range (overall 10-150 μm), consistent with the average values and suitable for exudate management and cell infiltration. The marked pore size reduction in AOEF@AP5 relative to AOE@AP5 is consistent with additional Schiff-base crosslinking introduced by F-SAP, which increases the effective crosslink density and tightens the network. Likewise, AOEF@AP5 showed smaller and denser pores than AOEF@AP1, in line with higher APF content. In AOEF@AP10, the mean pore size was slightly larger than in AOEF@AP5, likely reflecting a balance between increased amine content and partial loss of effective crosslinking when excess APF/PLLA is present.

All hydrogels showed an initial mass increase followed by a gradual decrease (**Fig. 1E**). During the swelling stage, mass increased until ~57 h, after which the hydrogels reached equilibrium. The equilibrium swelling ratios were relatively low and comparable among groups (**Fig. S11**) from 30.80% (AOE@AP5) to 28.24% (AOEF@AP1), 26.53% (AOEF@AP5) and 33.93% (AOEF@AP10). This moderate swelling capacity is consistent with the dense, highly crosslinked networks and is advantageous for avoiding wound maceration while still allowing effective exudate uptake. What is worth discussing is that after the PLLA fiber content of AOEF@AP10 increased significantly, though PLLA itself is relatively hydrophobic, excessive fibers hinder the uniform formation of dynamic crosslinking networks. This restricts the effective contact between part of the reactive groups and thus reduces the effective crosslinking density of the hydrogel network. Consequently, the equilibrium swelling ratio of AOEF@AP10 is higher than that of AOEF@AP1 and AOEF@AP5. Beyond ~57 h, mass loss dominated, indicating the onset of bulk degradation. All hydrogels degraded completely by ~250 h. The average degradation rates (**Fig. S11**) were 2.54 mg/h (AOE@AP5), 3.60 mg/h (AOEF@AP1), 3.01 mg/h (AOEF@AP5), and 3.33 mg/h (AOEF@AP10). The slightly slower degradation of AOEF@AP5 compared with AOE@AP5 is consistent with its higher dynamic crosslink density.

**Supplementary notes 4: Adhesion Strength and Electrical Conductivity of Hydrogels**

Aldehyde groups in the hydrogel precursor react with surface amino groups on skin, while catechol groups form multiple physical interactions with skin interface^4^, providing stable in situ adhesion. Lap-shear tests on fresh porcine skin showed that all formulations exhibited strong tissue adhesion (**Fig. 1J**). AOE@AP5 had an adhesion strength of 23.76 kPa, whereas peptide-loaded hydrogels showed slightly higher values: 25.38 kPa (AOEF@AP1), 26.60 kPa (AOEF@AP5) and 25.57 kPa (AOEF@AP10). This enhancement is consistent with additional covalent interactions between F-SAP and OSA-PBA, enhancing the cohesion of the hydrogel. Macroscopic tests further confirmed adhesion robustness. AOEF@AP5 remained firmly attached to pig skin during bending, folding, twisting (**Fig. S17**, **Fig. S18A, B, Movie S1**) and even under high-pressure water rinsing (**Movie S2**), supporting the stable application in dynamic wound sites.

All hydrogels exhibited conductivities in the range of 0.036–0.048 S/m (**Fig. S19**), comparable to reported values for skin tissue^5^. AOE@AP5 showed the lowest conductivity (0.036 S/m). Incorporation of F-SAP modestly increased conductivity to 0.042 S/m (AOEF@AP1), 0.038 S/m (AOEF@AP5) and 0.048 S/m (AOEF@AP10). This trend is attributed to ionizable amino acid residues within F-SAP, which generate additional mobile ions in aqueous media and slightly enhance ionic transport. What is worth discussing is that the PLLA fibers act as local mechano-electrical transducers: when subjected to biomechanical deformation, they generate piezoelectric charges or transient electrical potentials. The surrounding conductive hydrogel matrix then allows these locally generated electrical cues to be distributed more effectively across the hydrogel-tissue interface rather than remaining confined around individual fibers. In other words, the conductive hydrogel network may collect, transmit, and spatially homogenize the piezoelectric signals generated by PLLA fibers, thereby amplifying their biological relevance at the wound site.

**Supplementary notes 5: differences and distinct characteristics between Schiff base bonds and acylimide bonds in the hydrogel system.**

Schiff base bonds are relatively fast-forming and dynamically reversible, which contributes to rapid network formation, self-healing, injectability, and interfacial adhesion. Therefore, they mainly act as flexible and exchangeable crosslinking sites that allow the hydrogel to dynamically rearrange under deformation and maintain close contact with the wound surface. In contrast, acylhydrazone bonds are formed between aldehyde groups and hydrazide groups on AHA. Owing to the adjacent acylhydrazide structure and resonance stabilization, they generally exhibit higher hydrolytic stability than ordinary Schiff base imine bonds while still retaining dynamic reversibility. Thus, acylhydrazone bonds function as more stable dynamic crosslinking points. They help maintain the integrity, mechanical robustness, and durability of the hydrogel under the moist wound environment. Together with phenylboronate ester bonds, these interactions construct a multi-dynamic covalent hydrogel network suitable for the complex and dynamic wound microenvironment.

**Reference**

1. Tong J, Yang F, Li X, Xu X, Wang GX. Mechanical Characterization and Material Modeling of Diabetic Aortas in a Rabbit Model. *Annals of biomedical engineering* **46**, 429-442 (2018).

2. Vinikoor T*, et al.* Injectable and biodegradable piezoelectric hydrogel for osteoarthritis treatment. *Nature communications* **14**, 6257 (2023).

3. Le TT*, et al.* Piezoelectric Nanofiber Membrane for Reusable, Stable, and Highly Functional Face Mask Filter with Long-Term Biodegradability. **32**, 2113040 (2022).

4. Ouyang C*, et al.* One-Step Formed Janus Hydrogel with Time-Space Regulating Properties for Suture-Free and High-Quality Tendon Healing. *Advanced science (Weinheim, Baden-Wurttemberg, Germany)* **12**, e2411400 (2025).

5. Luo J*, et al.* Piezoelectric dual-network tough hydrogel with on-demand thermal contraction and sonopiezoelectric effect for promoting infected-joint-skin-wound healing via FAK and AKT signaling pathways. *National science review* **12**, (2025).
